# Supplementary figures and images for: A structurally conserved site in AUP1 binds the E2 enzyme UBE2G2 and is essential for ER-associated degradation
Source: PLoS Biol. 2021 Dec 8;19(12):e3001474. doi: 10.1371/journal.pbio.3001474 (PMC8699718; doi:10.1371/journal.pbio.3001474)

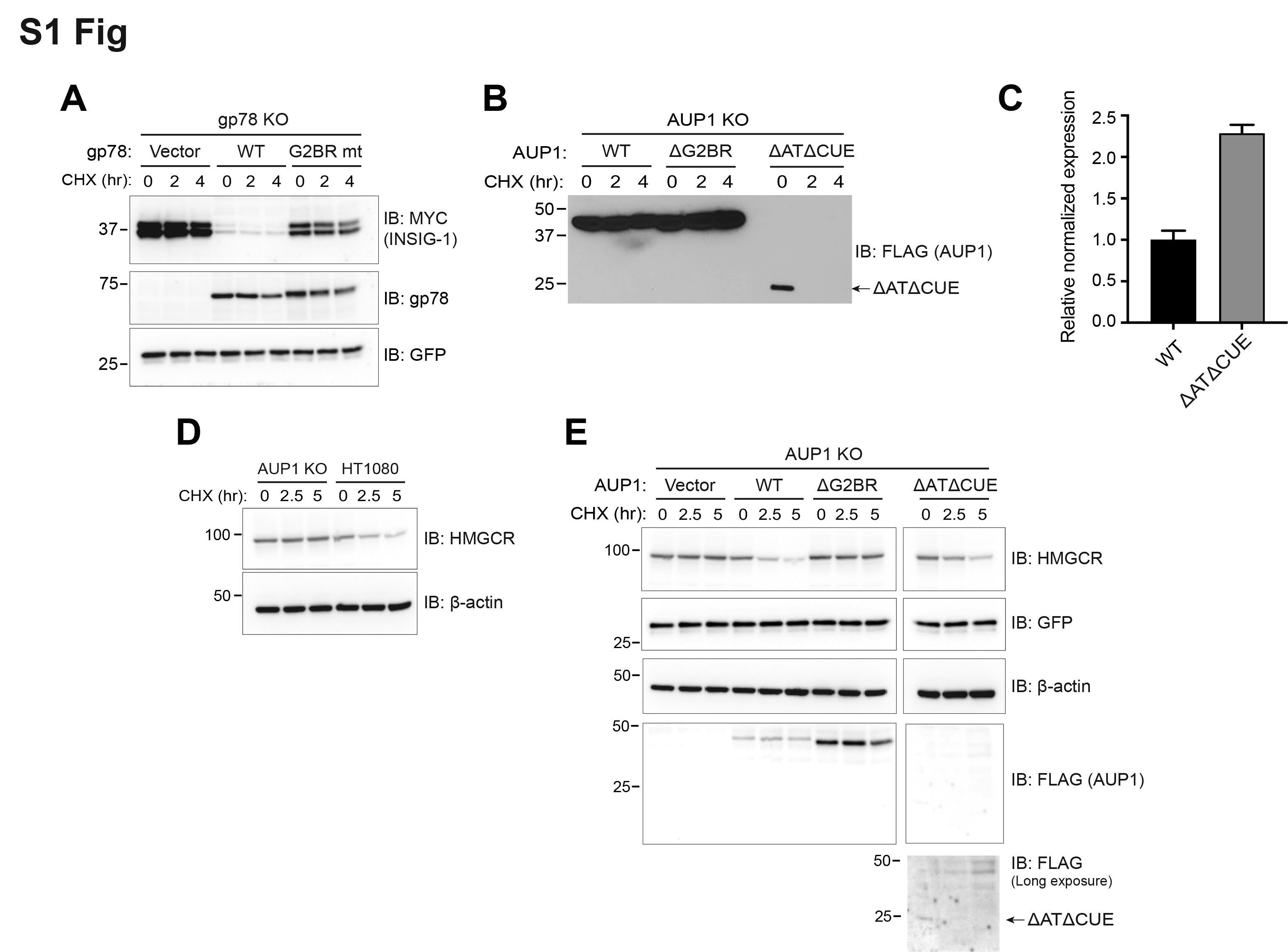

Supplement: S1 Fig — (A) gp78 KO cells were transfected with plasmid encoding WT or G2BR mutant gp78 and MYC-tagged INSIG-1, and assessed for INSIG-1 degradation by CHX chase. (B) Long exposure of FLAG immunoblot from Fig 1F demonstrating the relatively low expression of the AUP1 ΔATΔCUE mutant. (C) AUP1 KO cells were transfected with plasmids encoding FLAG-tagged AUP1 WT or ΔATΔCUE mutant and transcript levels determined following isolation of RNA from cells and qPCR to amplify the FLAG-tagged constructs. Expression of the ΔATΔCUE mutant is presented relative to WT AUP1. Mean and standard deviation are shown. (D) HT1080 parental and AUP1 KO cells were allowed to accumulate HMGCR in LPDS media for 24 hours. Basal degradation of HMGCR was assessed by addition of CHX in LPDS media. (E) HT1080 AUP1 KO cells were transfected with the indicated plasmids and switched to LPDS media 36 hours post-transfection. After 24 hours, cells were treated with CHX in LPDS media to assess basal degradation of HMGCR. The data underlying this figure can be found in S2 and S3 Data. AUP1, ancient ubiquitous protein 1; CHX, cycloheximide; G2BR, UBE2G2 Binding Region; HMGCR, 3-hydroxy-3-methylglutaryl CoA reductase; KO, knockout; LPDS, lipoprotein-deficient serum; qPCR, quantitative polymerase chain reaction; WT, wild type. (TIF) [file pbio.3001474.s001.tif]

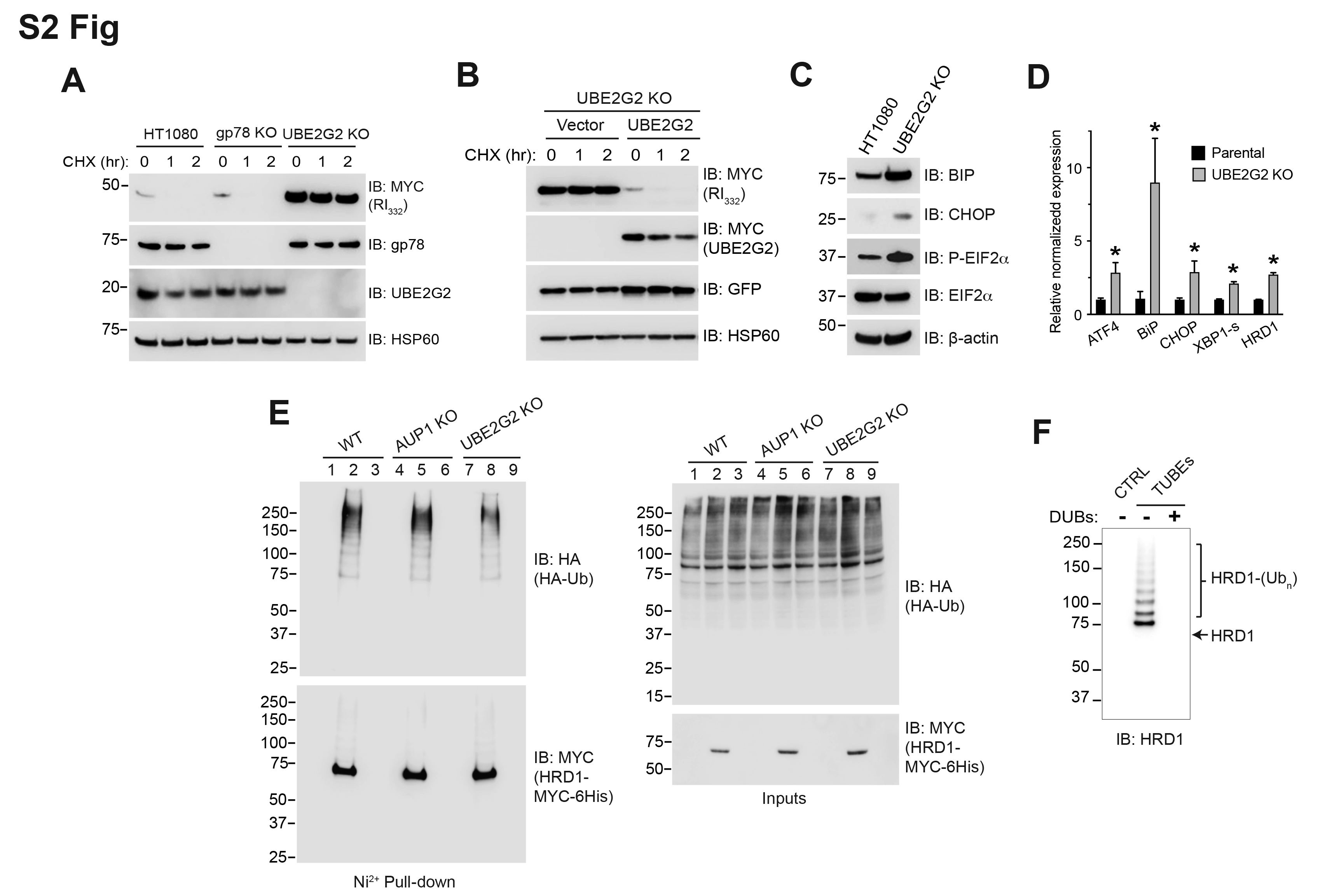

Supplement: S2 Fig — (A) HT1080 or the indicated KO cells were transfected with plasmid encoding RI332-MYC and degradation assessed by CHX chase. (B) HT1080 UBE2G2 KO cells were transfected with RI332-MYC and with either vector or MYC-UBE2G2. RI332 stability was assessed as in (A). (C) HT1080 and UBE2G2 KO cells were assessed for indicators of an ER stress response by western blot. Total EIF2α and actin serve as internal controls. (D) HT1080 parental and UBE2G2 KO cells were assessed for relative transcript levels of ER stress markers by qPCR. For each marker, expression is presented relative to HT1080. Mean and standard deviation are shown (*P < 0.05). (E) (Left panel) indicated cells were transfected with plasmid encoding HA-ubiquitin and either empty vector (lanes 1, 4, and 7) or HRD1-MYC-6His (lanes 2, 5, and 8), lysed in urea buffer and pulled down with nickel (Ni2+) beads. The supernatant was then subjected to a second pull-down (lanes 3, 6, and 9) with nickel beads to confirm the efficiency of the first pull-down. Eluted samples were immunoblotted for HA-ubiquitin and HRD1-MYC-6His. Inputs of vector transfected (lanes 1, 4, and 7), HRD1-MYC-6His transfected (lanes 2, 5, and 8), and second pull-down (lanes 3, 6, and 9) are shown in the right panel. (F) Ubiquitinated proteins were enriched with a mixture of TUBE1 and TUBE2 agarose and treated with buffer or a cocktail of deubiquitinating enzymes. Agarose (UM400) beads served as a control. After extensive washing, proteins were eluted with 2X SDS sample buffer and resolved by SDS-PAGE and immunoblotted for HRD1. The data underlying this figure can be found in S2 and S4 Data. CHX, cycloheximide; KO, knockout; qPCR, quantitative polymerase chain reaction. (TIF) [file pbio.3001474.s002.tif]

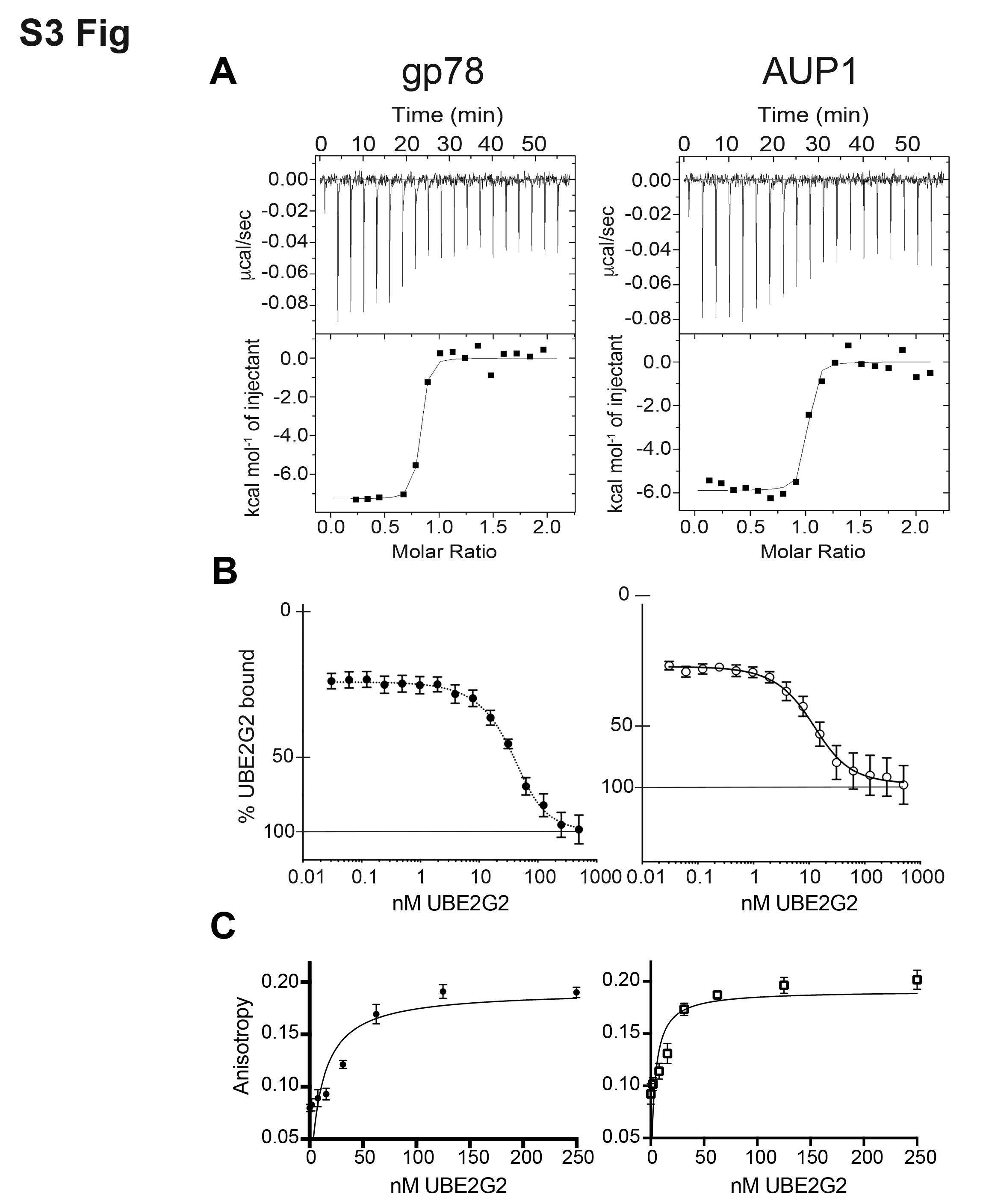

Supplement: S3 Fig — (A) ITC titration curves from experiment performed with purified UBE2G2 and G2BRgp78 (left) or G2BRAUP1 (right) peptides. (B) FITC-labeled G2BRgp78 or G2BRAUP1 peptides were incubated with increasing concentrations of purified UBE2G2 at 22°C, and binding was assessed by MST to determine dissociation constants (Kd) between the G2BR and UBE2G2. (C) Binding was assessed as in (B) using FP. The data underlying this figure can be found in S5–S7 Data. AUP1, ancient ubiquitous protein 1; FP, fluorescence polarization; G2BR, UBE2G2 Binding Region; ITC, isothermal titration calorimetry; MST, microscale thermophoresis. (TIF) [file pbio.3001474.s003.tif]

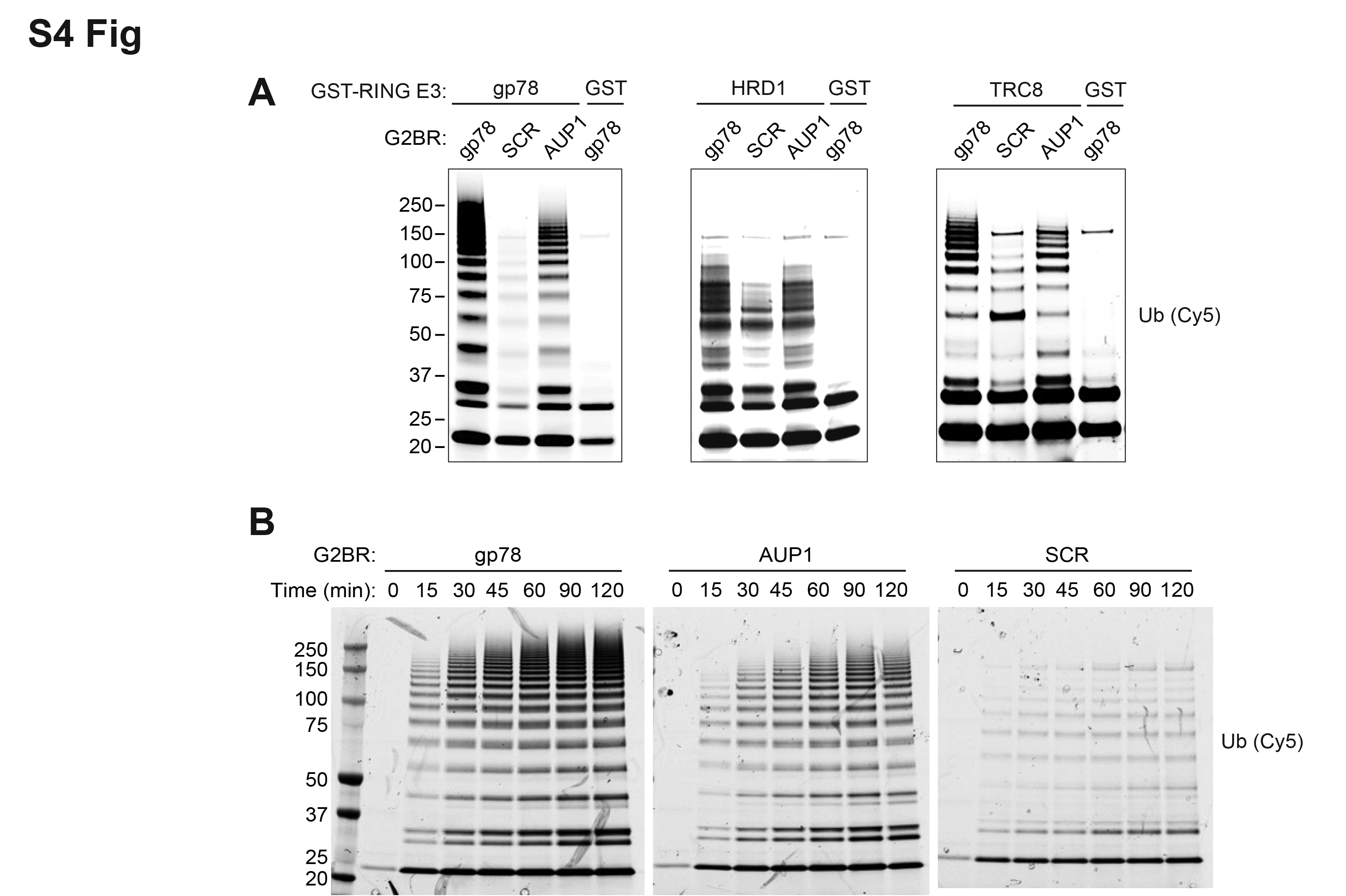

Supplement: S4 Fig — (A) GST fusions to the RING domain of indicated E3s were incubated with E1, UBE2G2, Cy5-labeled ubiquitin, and either a scrambled G2BR (SCR), G2BRgp78, or G2BRAUP1 peptide for 1.5 hours at 37°C. Reactions were resolved by SDS-PAGE and Cy5-ubiquitin was visualized on a phosphorimager. (B) Ubiquitination assays described in (A) were carried out with GST-gp78 RING for the indicated times with the specified G2BR peptides. The data underlying this figure can be found in S2 Data. AUP1, ancient ubiquitous protein 1; G2BR, UBE2G2 Binding Region. (TIF) [file pbio.3001474.s004.tif]

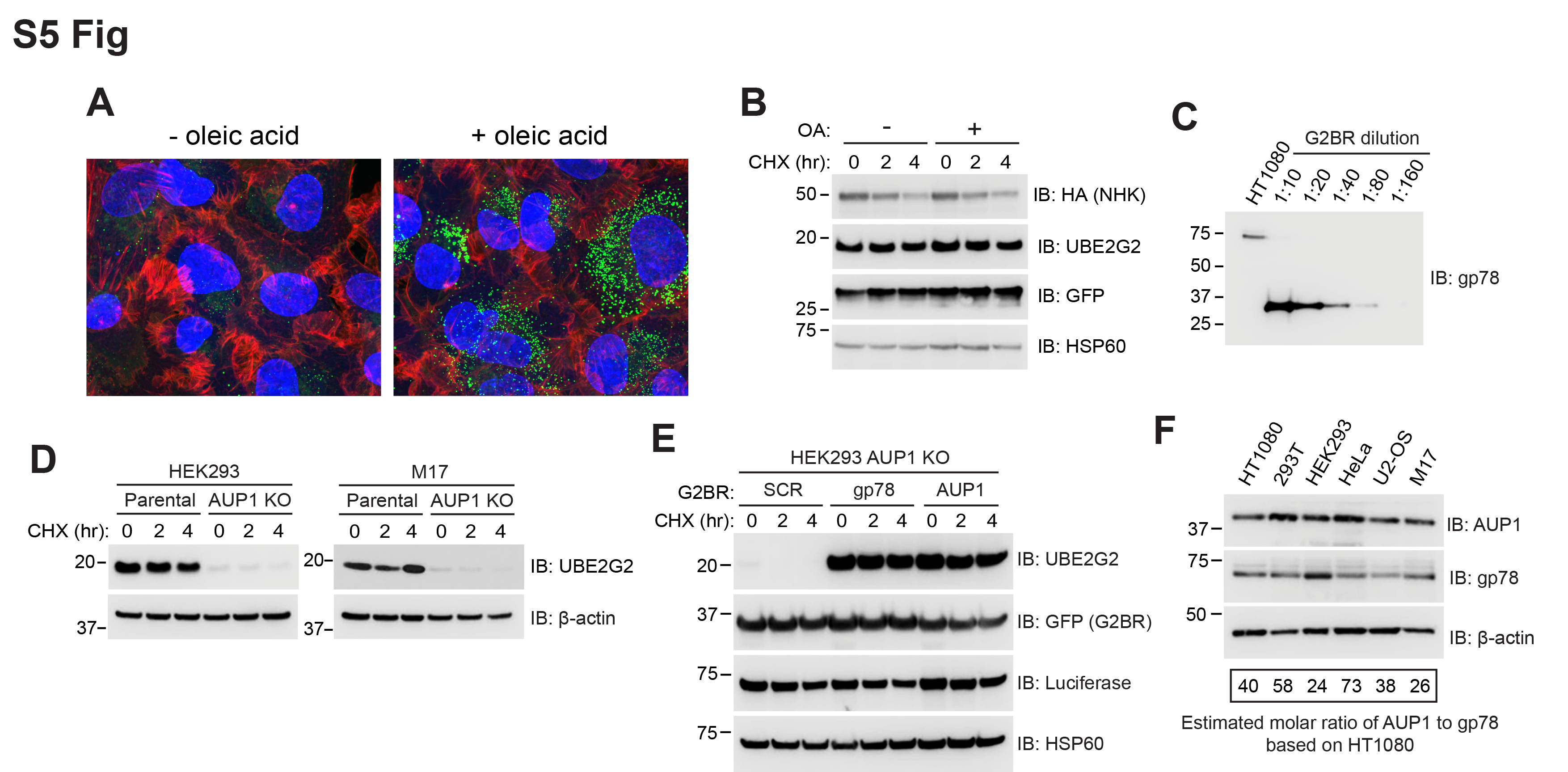

Supplement: S5 Fig — (A) HT1080 cells were treated with 200 μM oleic acid (OA) overnight prior to fixation and staining to visualize lipid droplets (green), filamentous actin (red), and nuclei (blue) by confocal microscopy. Maximum intensity projection images (without deconvolution) of representative fields are shown. (B) HT1080 cells were treated with oleic acid as in (A) and assessed for degradation of transfected NHK-HA and endogenous UBE2G2. (C) To determine expression of GFP-G2BR fusions in transfection experiments, AUP1 KO cells were transfected with plasmid encoding GFP-G2BRgp78 and dilutions of lysate were resolved by SDS-PAGE alongside HT1080 lysate. Detection was carried out with affinity-purified antibodies directed against the G2BR of gp78 (Ab2) and compared to levels of endogenous gp78. The level of transfected GFP-G2BR is approximately 40-fold that of endogenous gp78. (D) Levels of UBE2G2 were monitored by CHX chase in HEK293 (left panel) and M17 (right panel) parental and AUP1 KO cells. (E) HEK293 AUP1 KO cells were transfected with plasmids encoding MYC-UBE2G2 and GFP fusions of either a scrambled G2BR (SCR), G2BRgp78, or G2BRAUP1, and the turnover of UBE2G2 was assessed. (F) Approximately 1 × 10^4^ cell equivalents of the indicated cell lines were assessed for levels of AUP1 and gp78. Using the 40:1 ratio of the two proteins in HT1080 derived from Fig 7E, approximate relative levels of AUP1 to gp78 were calculated for each cell line. The data underlying this figure can be found in S2 Data. AUP1, ancient ubiquitous protein 1; CHX, cycloheximide; G2BR, UBE2G2 Binding Region; KO, knockout; NHK, Null Hong Kong. (TIF) [file pbio.3001474.s005.tif]

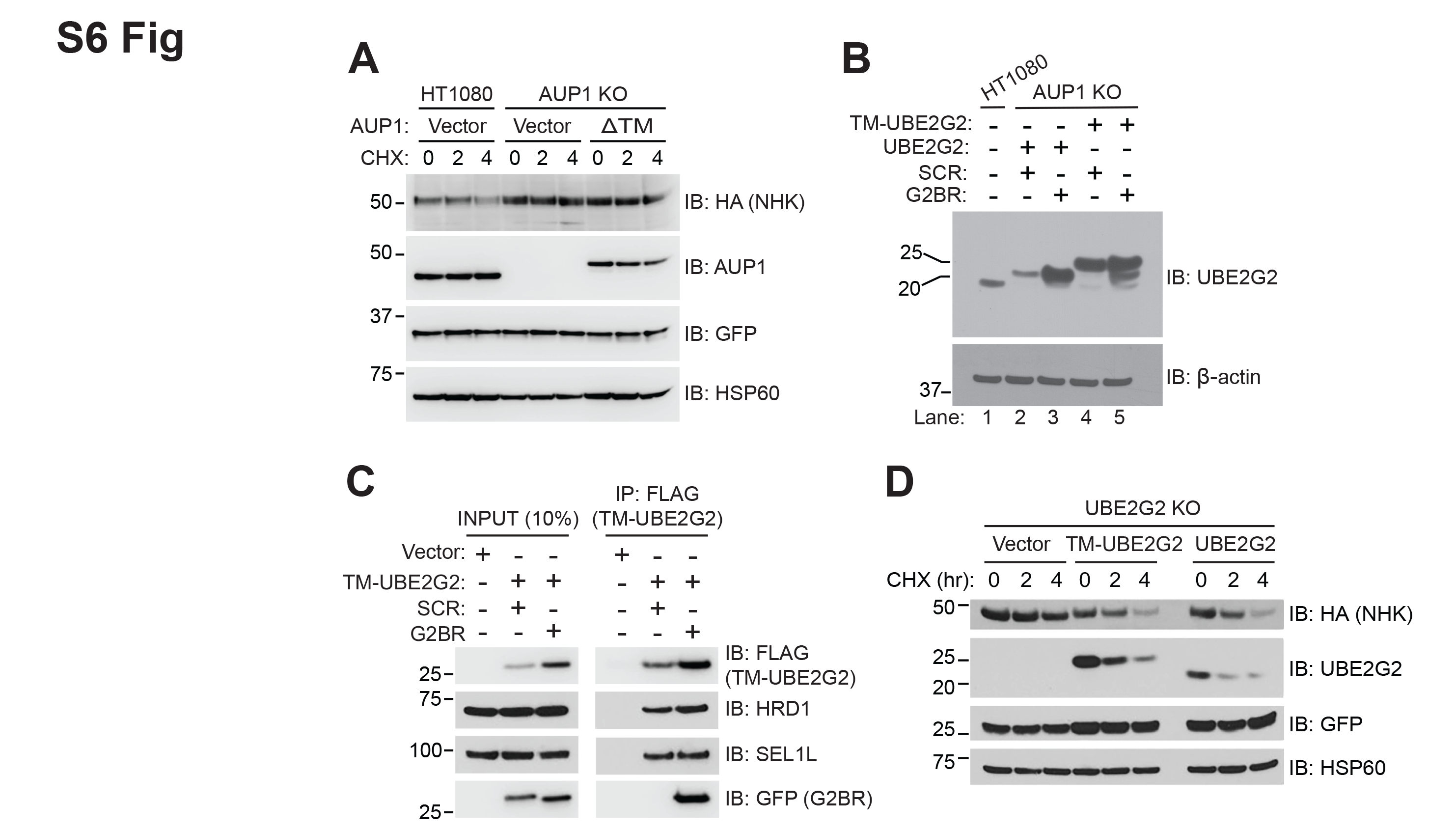

Supplement: S6 Fig — (A) HT1080 or AUP1 KO cells were transfected with NHK-HA and either empty vector or FLAG-tagged AUP1 lacking the hairpin transmembrane region (ΔTM), and NHK turnover assessed by CHX chase. (B) Relative levels of endogenous (HT1080) or overexpressed UBE2G2 from t = 0 time points in Fig 8C (lanes 1 to 3) and 8H (lanes 4 and 5) in which AUP1 KO cells were transfected with MYC-UBE2G2 or TM-UBE2G2 and GFP fusions of either a scrambled G2BR (SCR) or G2BRAUP1. (C) HT1080 cells were transfected with plasmid encoding TM-UBE2G2 to which a FLAG tag had been added at the carboxyl terminus to facilitate co-immunoprecipitation. Immunoprecipitates from digitonin lysates were blotted for associated HRD1 and SEL-1L. Inputs are shown on the left. (D) UBE2G2 KO cells were transfected with NHK-HA and either empty vector, MYC-UBE2G2 or TM-UBE2G2, and NHK turnover assessed by CHX chase. The data underlying this figure can be found in S2 Data. AUP1, ancient ubiquitous protein 1; CHX, cycloheximide; G2BR, UBE2G2 Binding Region; KO, knockout; NHK, Null Hong Kong. (TIF) [file pbio.3001474.s006.tif]
