# Supplementary material for: A structurally conserved site in AUP1 binds the E2 enzyme UBE2G2 and is essential for ER-associated degradation
Source: PLoS Biol. 2021 Dec 8;19(12):e3001474. doi: 10.1371/journal.pbio.3001474 (PMC8699718; doi:10.1371/journal.pbio.3001474)
Supplement: S1 Data — (PDF) [file pbio.3001474.s007.pdf]

Fig 1A

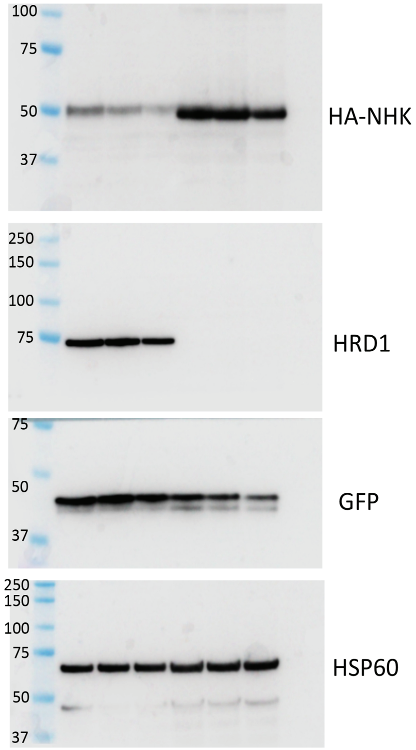

FIG 1B

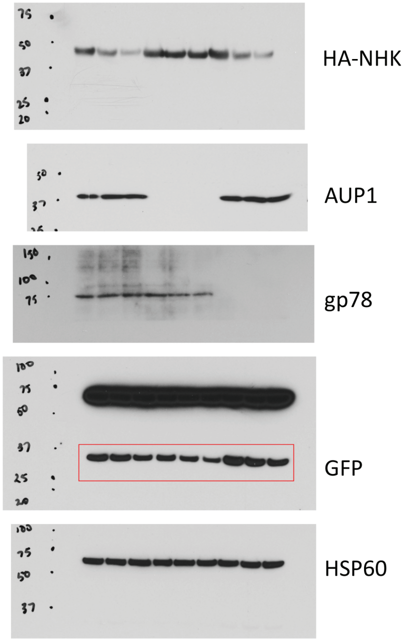

Fig 1C

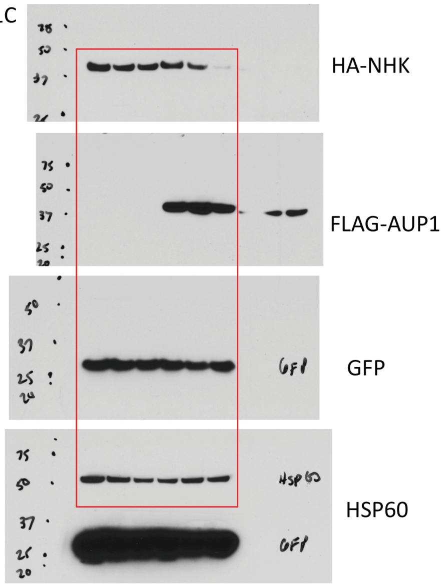

FIG 1D

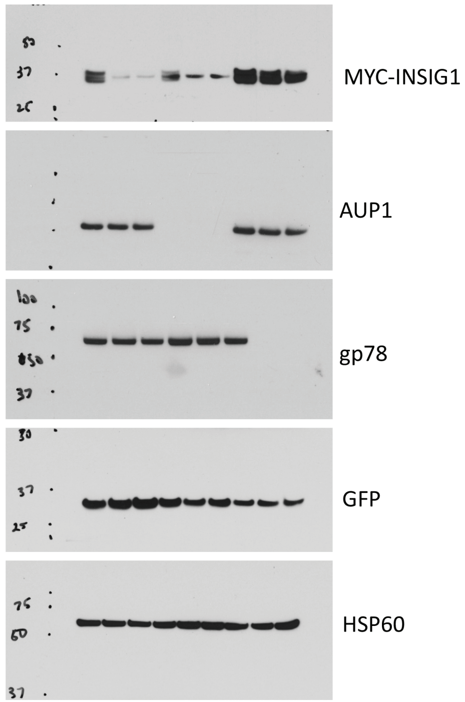

Fig 1F

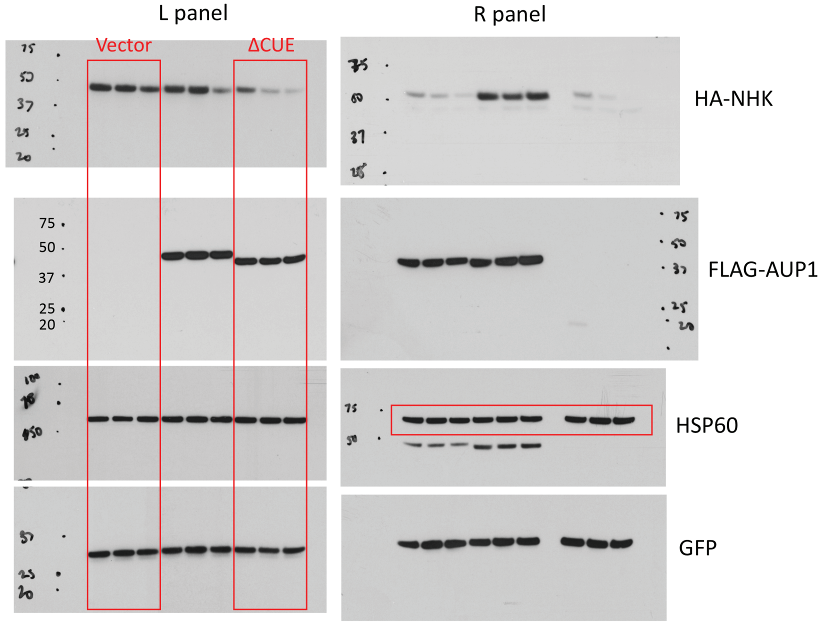

Fig 1G

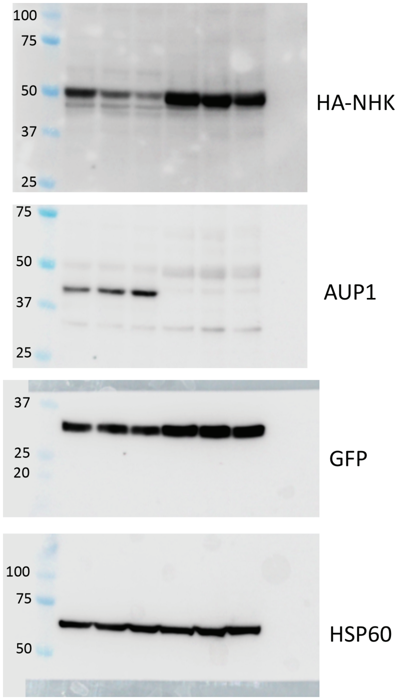

FIG 1H

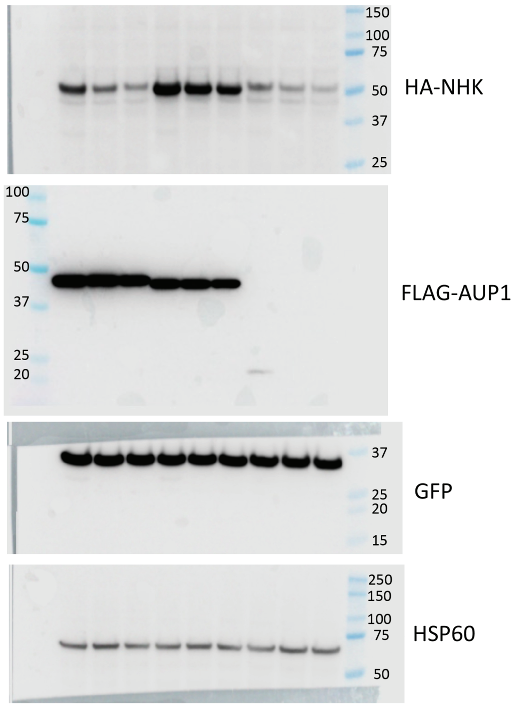

Fig 2A

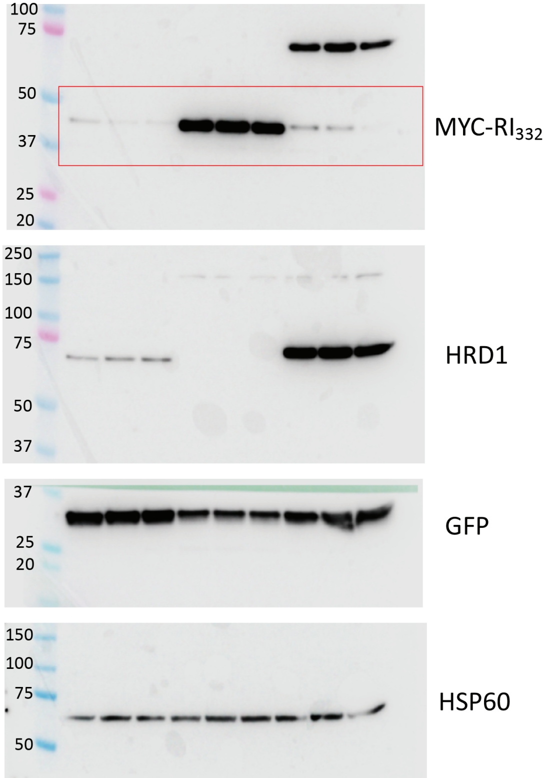

Fig 2B

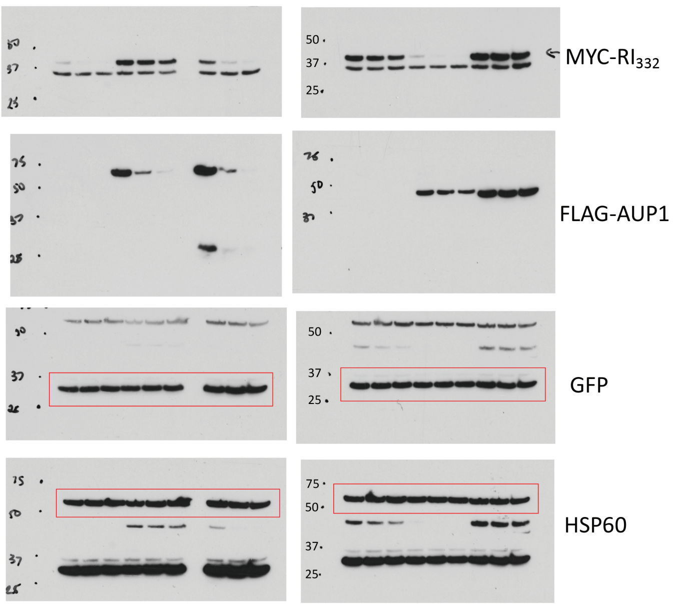

Fig 2C

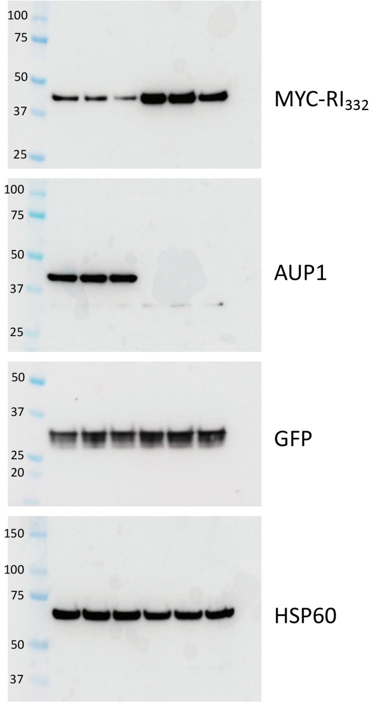

Fig 2D

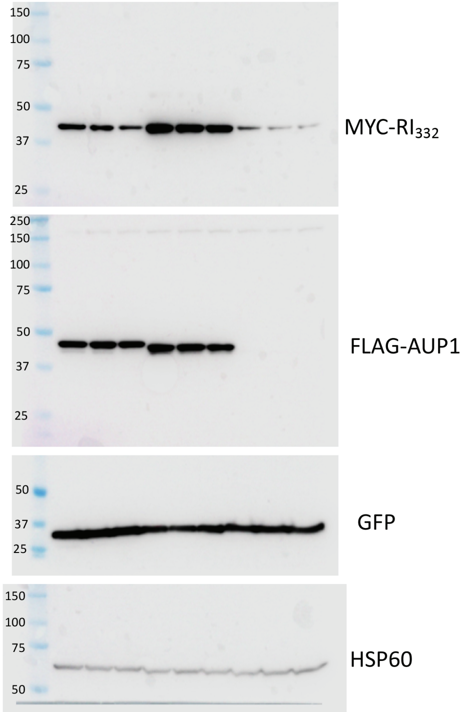

Fig 2E

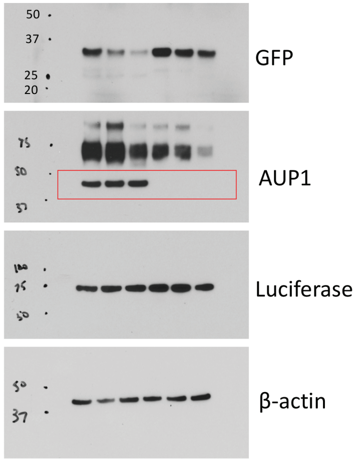

Fig 2F

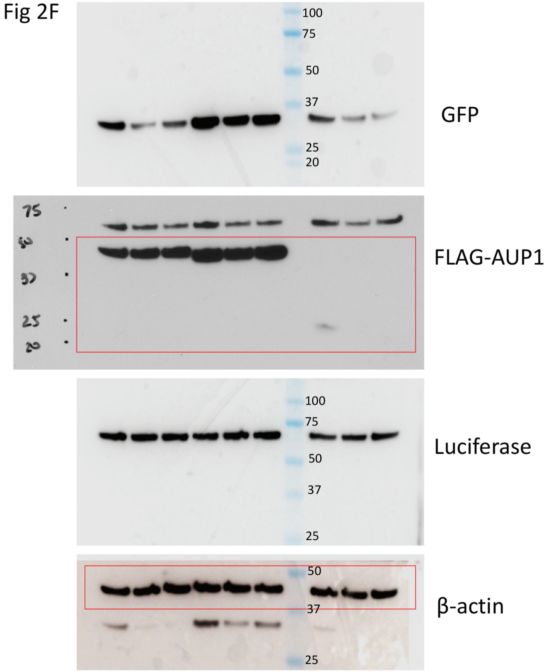

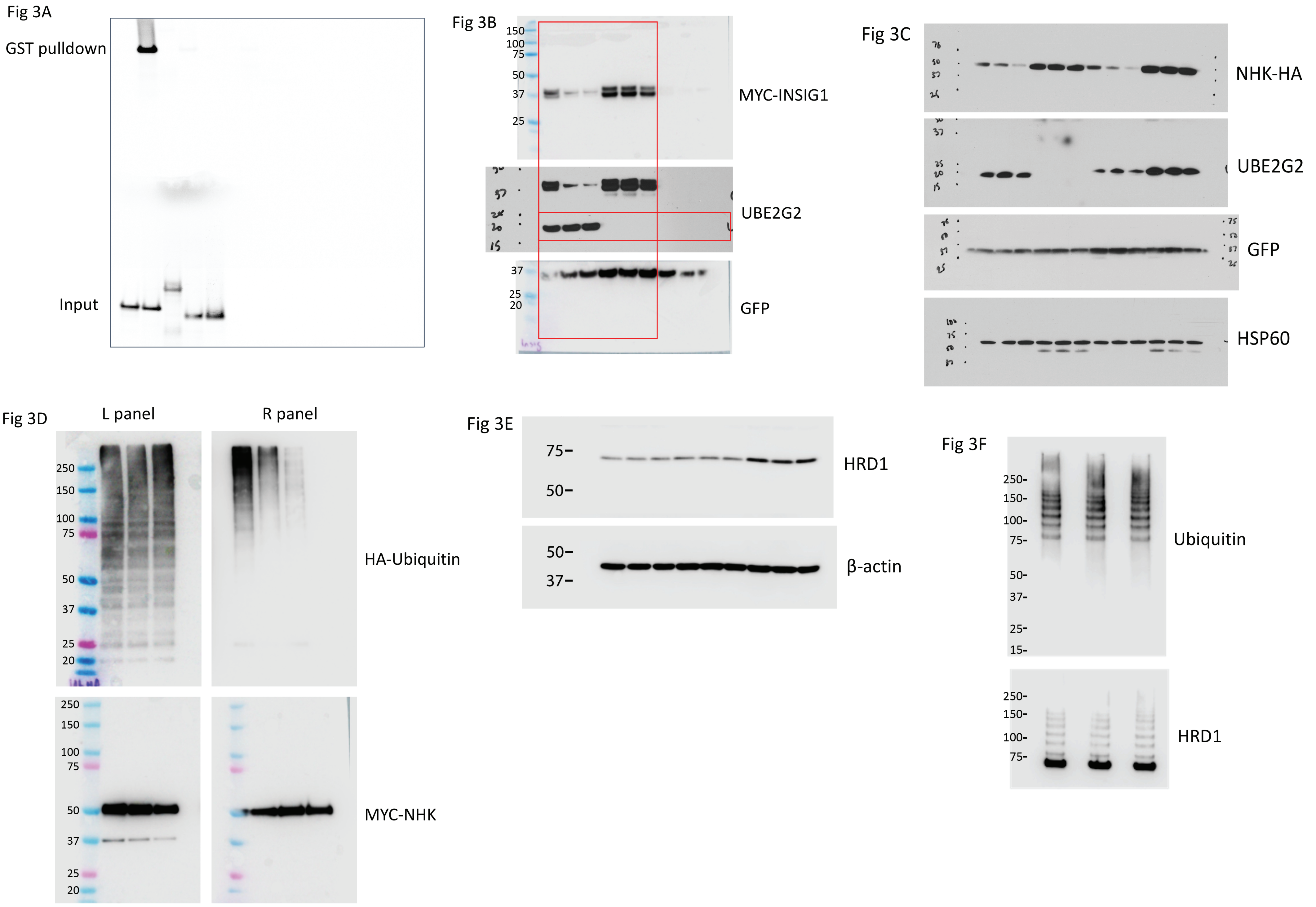
Fig 3A
GST pulldown
Input
Fig 3B
150
100
75
50
37
25
MYC-INSIG1
UBE2G2
37
25
20
GFP
Fig 3C
NHK-HA
UBE2G2
GFP
HSP60
Fig 3D
L panel
R panel
250
150
100
75
50
37
25
20
HA-Ubiquitin
250
150
100
75
50
37
25
20
MYC-NHK
Fig 3E
75
50
HRD1
50
37
β-actin
Fig 3F
250
150
100
75
50
37
25
15
Ubiquitin
250
150
100
75
HRD1

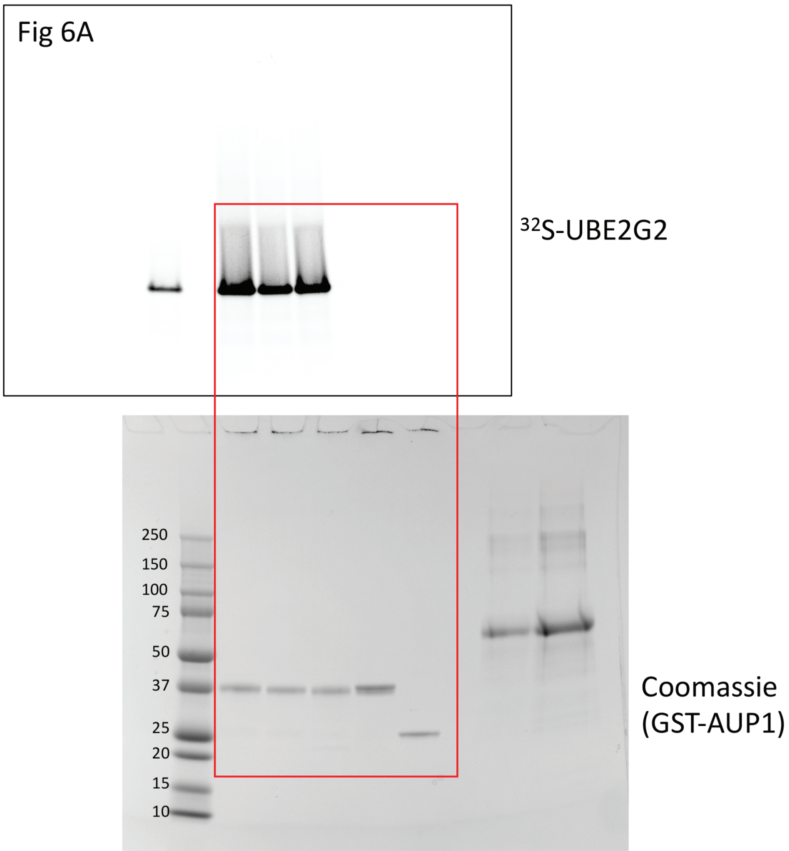
Fig 6A
^{32}S-UBE2G2
250
150
100
75
50
37
25
20
15
10
Coomassie
(GST-AUP1)

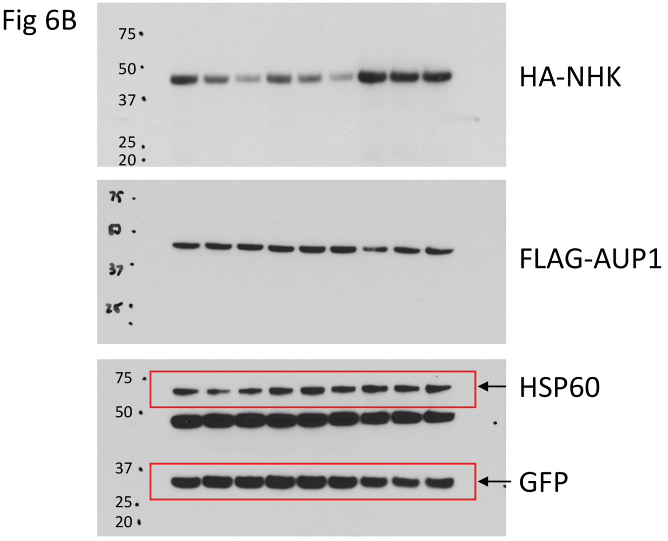
Fig 6B
HA-NHK
FLAG-AUP1
HSP60
GFP

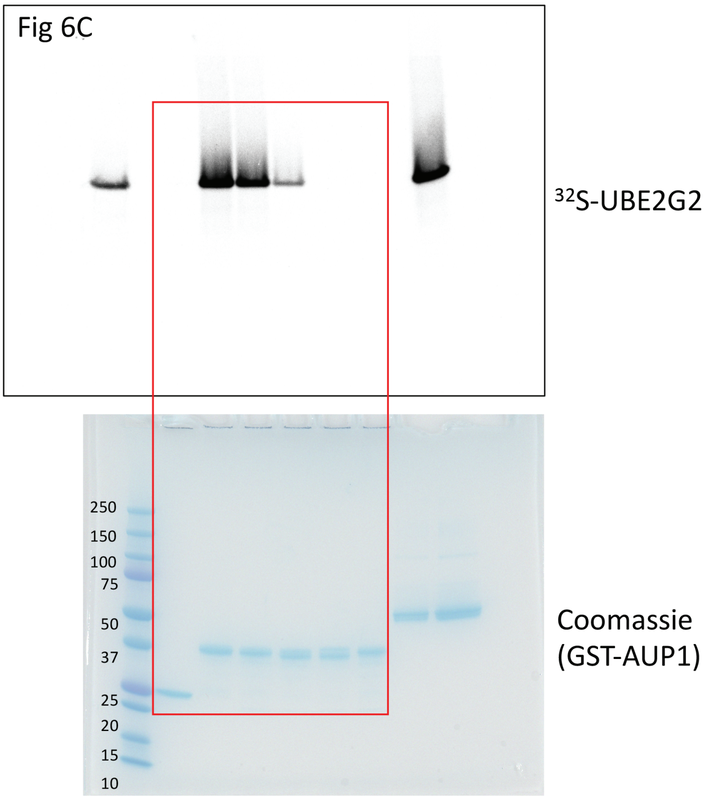
Fig 6C
^{32}S-UBE2G2
250
150
100
75
50
37
25
20
15
10
Coomassie
(GST-AUP1)

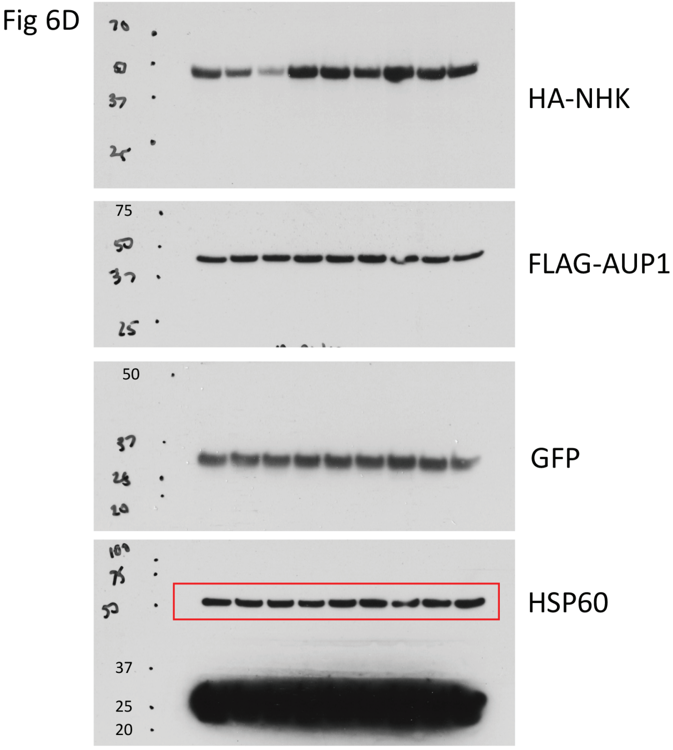
Fig 6D
HA-NHK
FLAG-AUP1
GFP
HSP60

Fig 7A

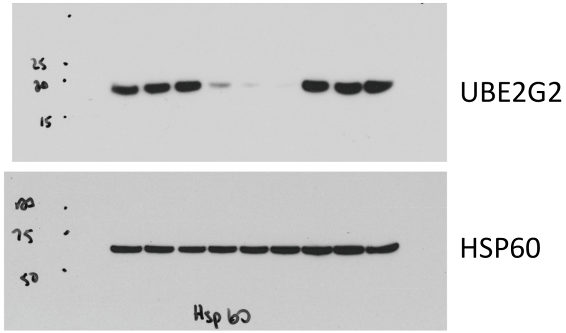

Fig 7C

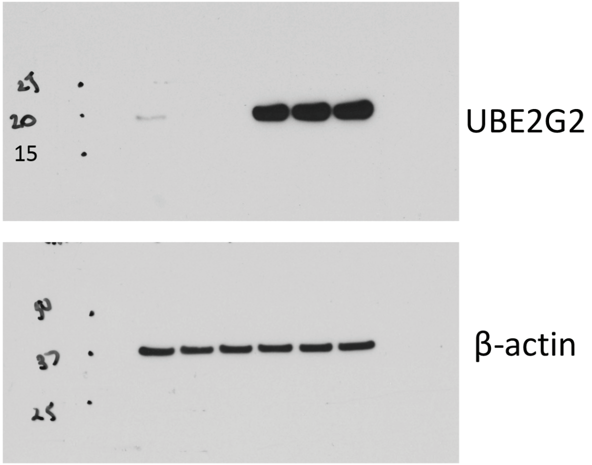

Fig 7D

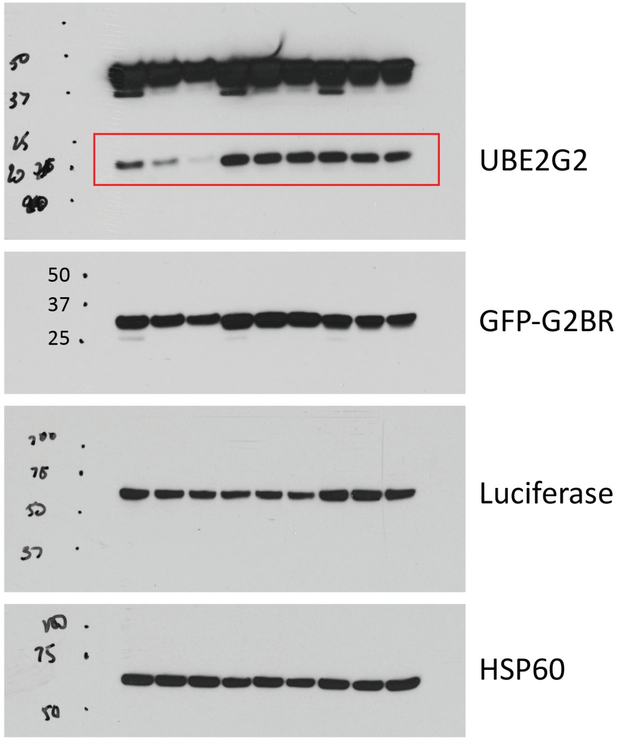

Fig 7E

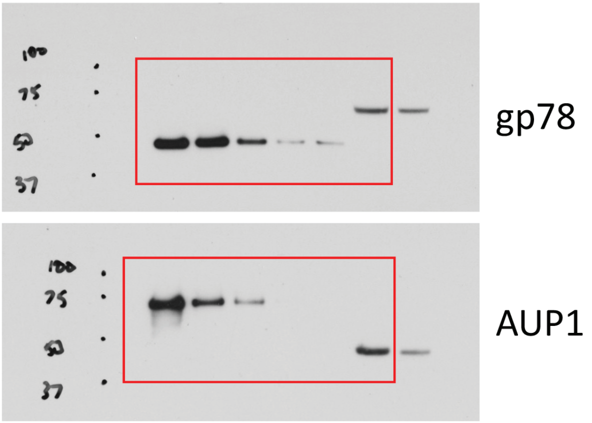

Fig 8A

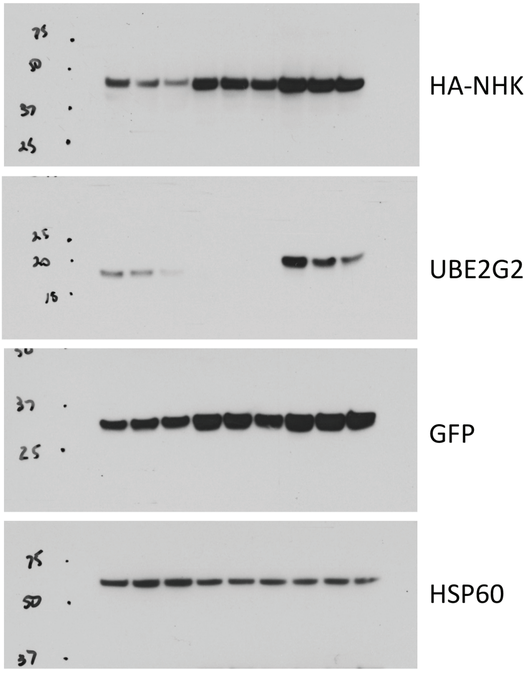

Fig 8B

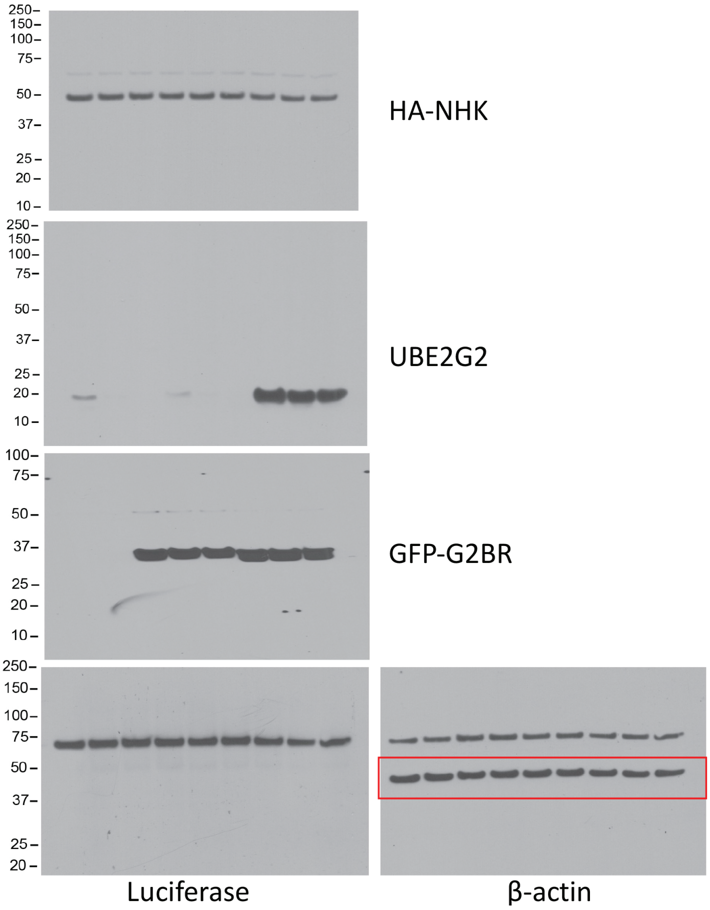

Fig 8C

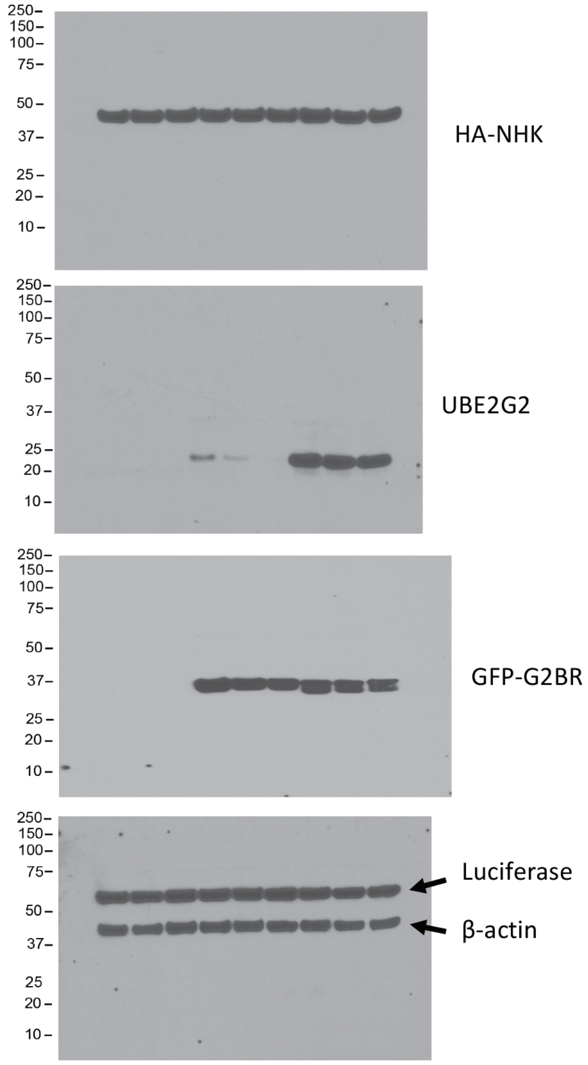

Fig 8D

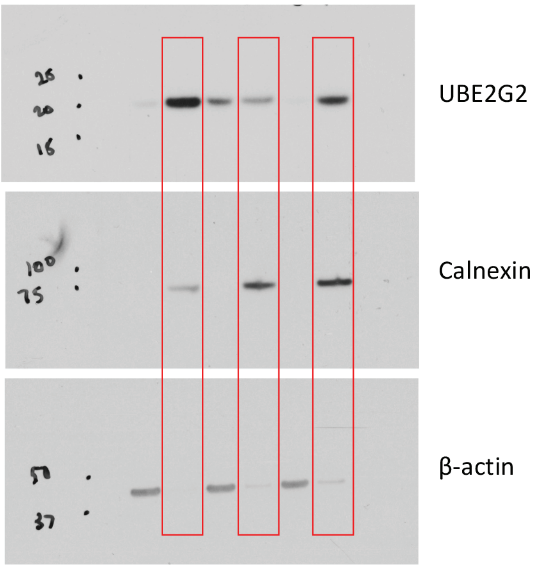

Fig 8E

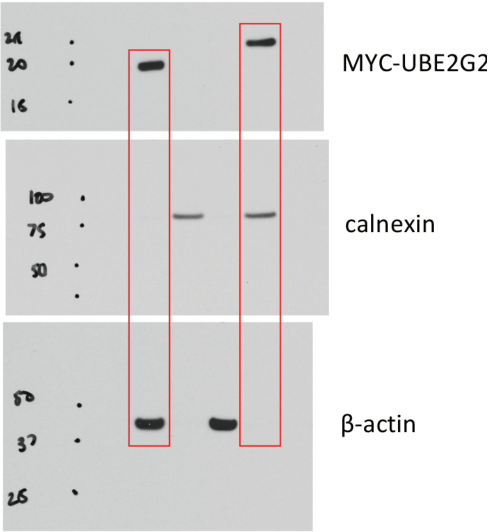

Fig 8H

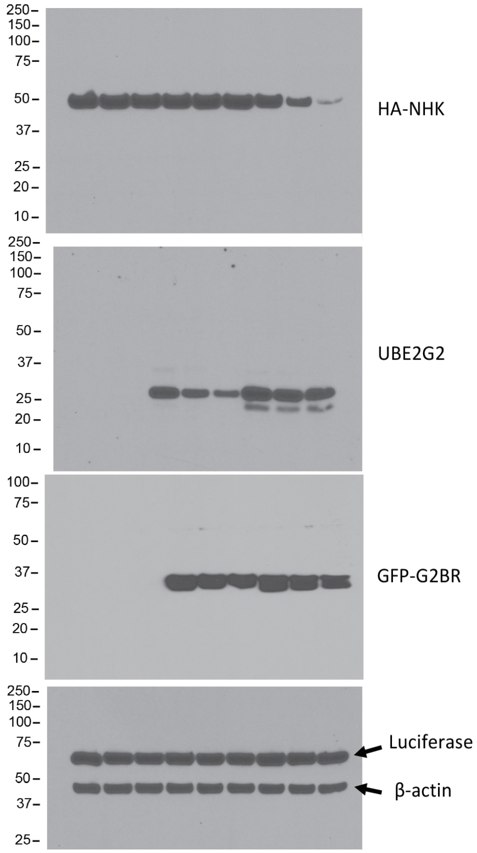
HA-NHK
UBE2G2
GFP-G2BR
Luciferase
β-actin

Fig 8I

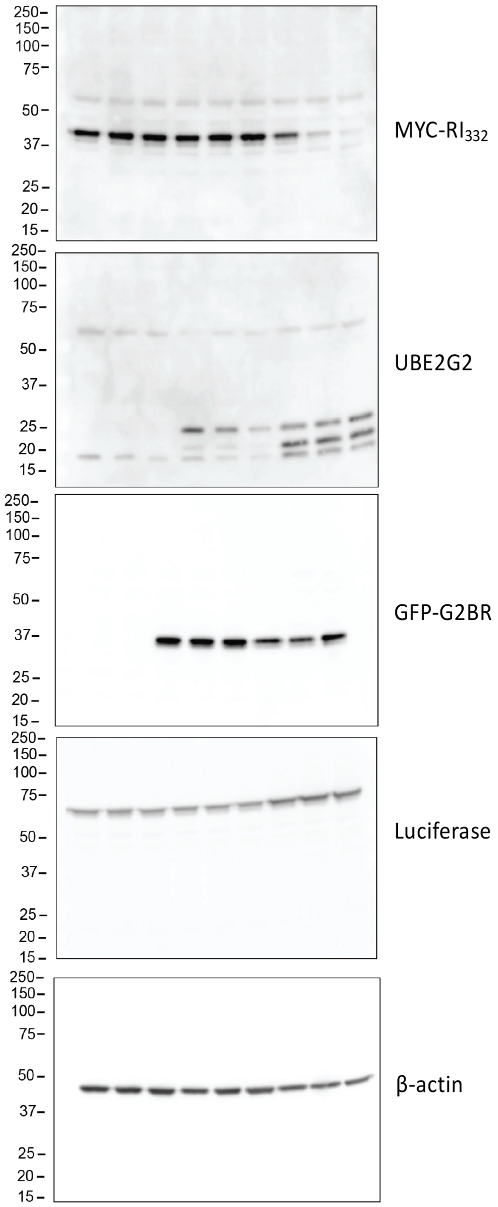
MYC-RI$_{332}$
UBE2G2
GFP-G2BR
Luciferase
β-actin
