# Supplementary material for: A structurally conserved site in AUP1 binds the E2 enzyme UBE2G2 and is essential for ER-associated degradation
Source: PLoS Biol. 2021 Dec 8;19(12):e3001474. doi: 10.1371/journal.pbio.3001474 (PMC8699718; doi:10.1371/journal.pbio.3001474)

S1A Fig

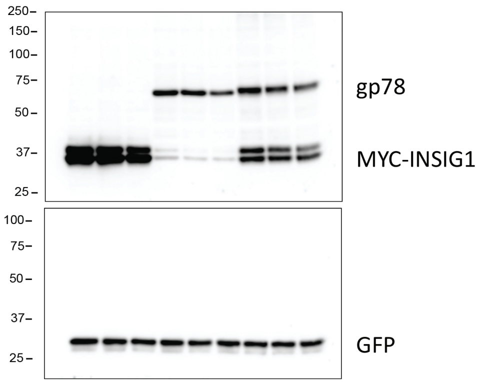

S1B Fig

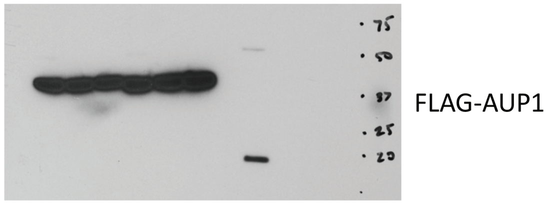

S1D Fig

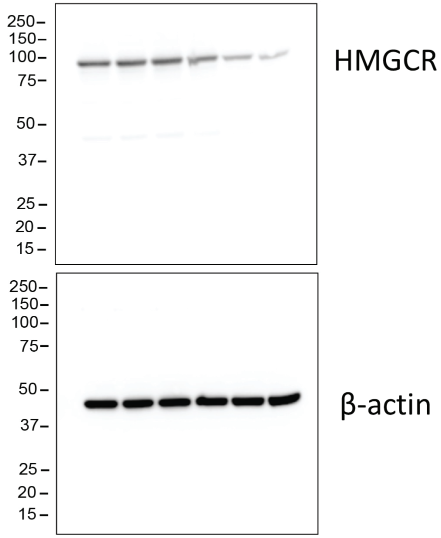

S1E Fig

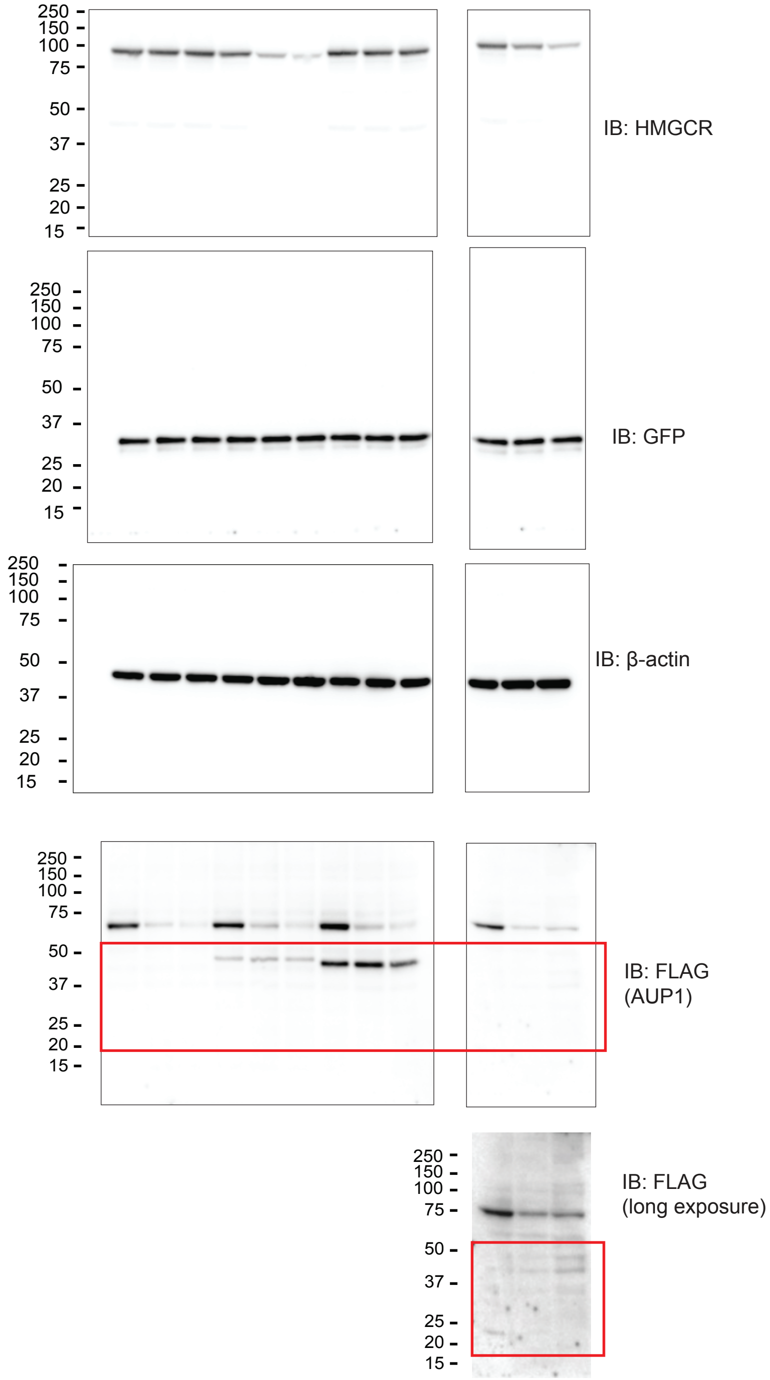

S2A Fig

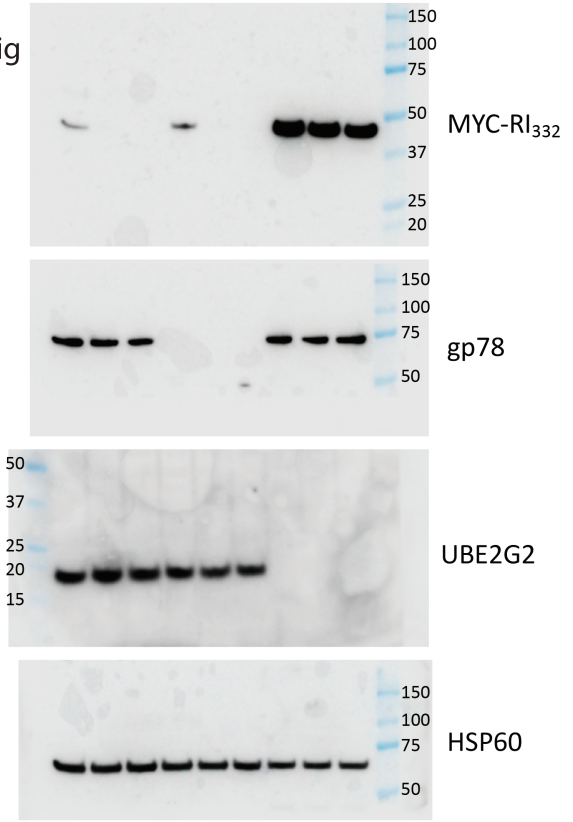

S2B Fig

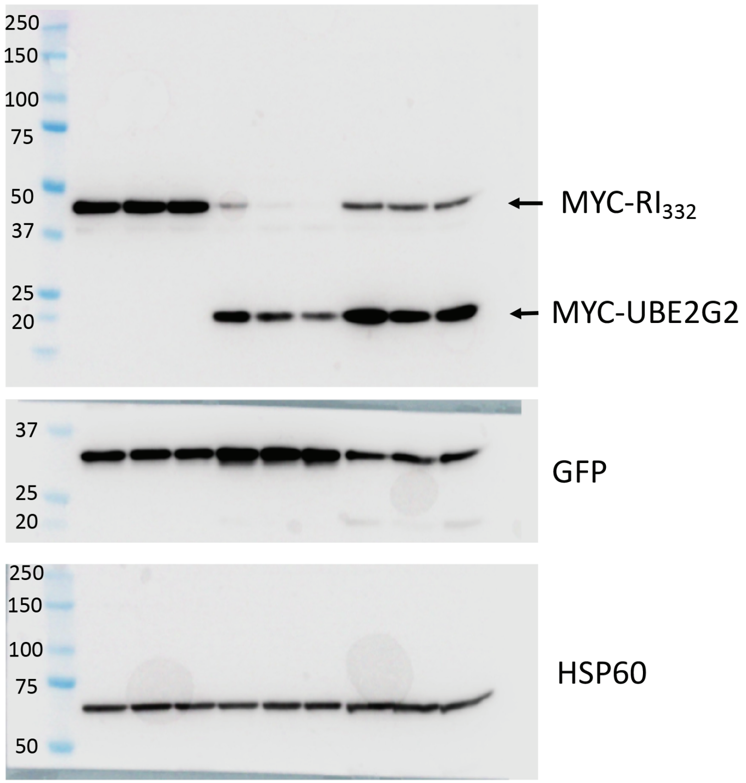

S2C Fig

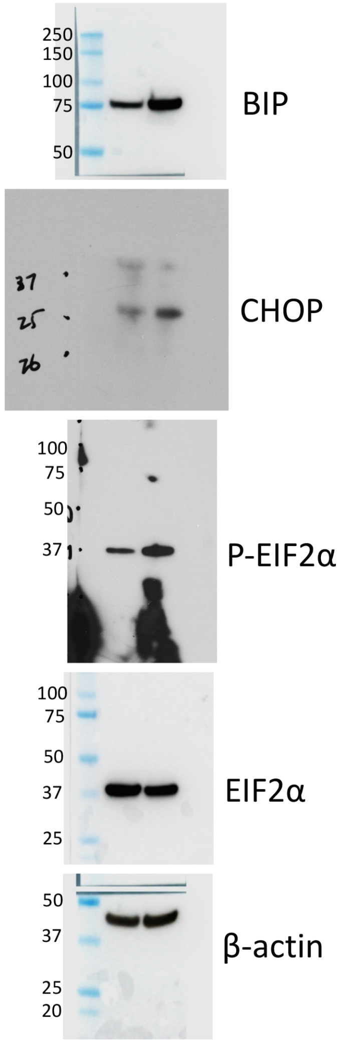

S2E Fig

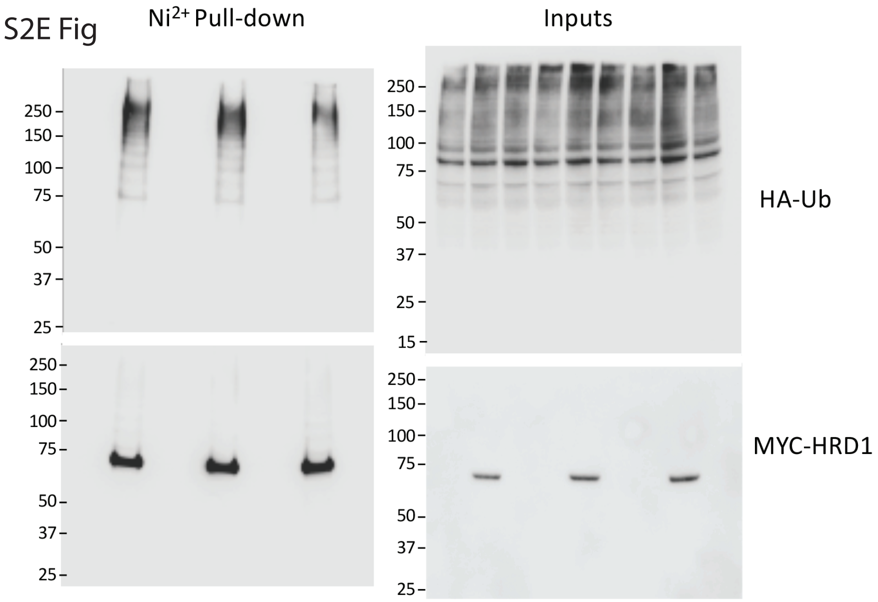

S2F Fig

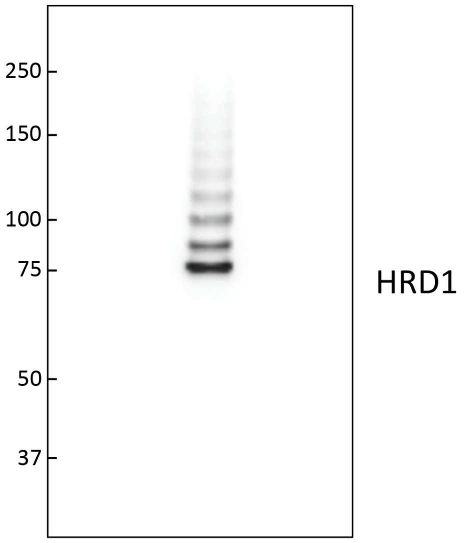

S4A Fig

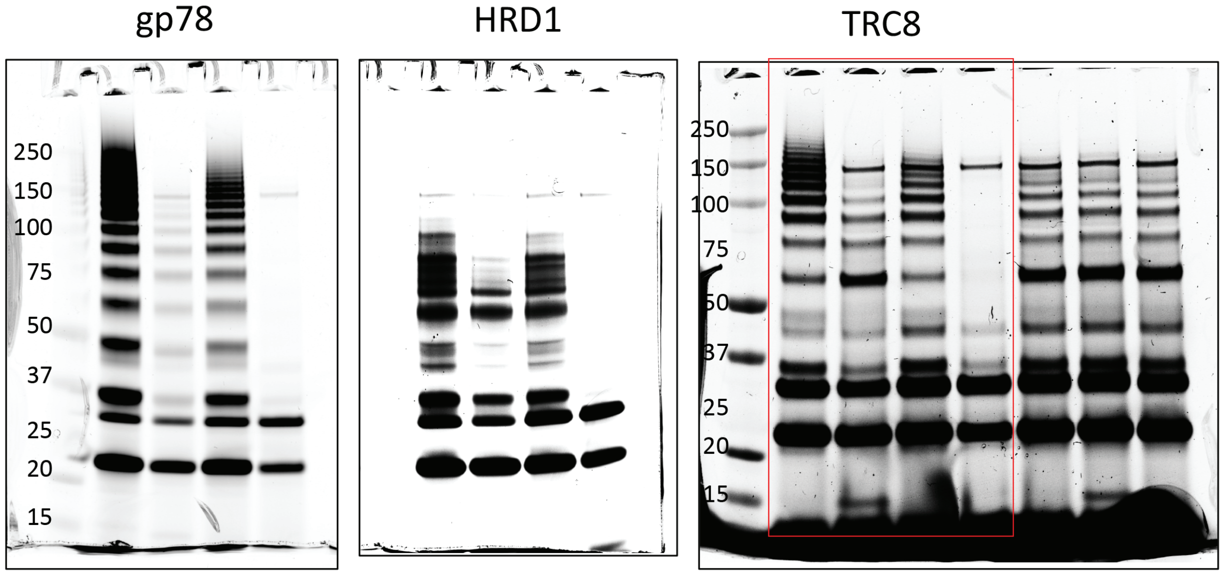

S4B Fig

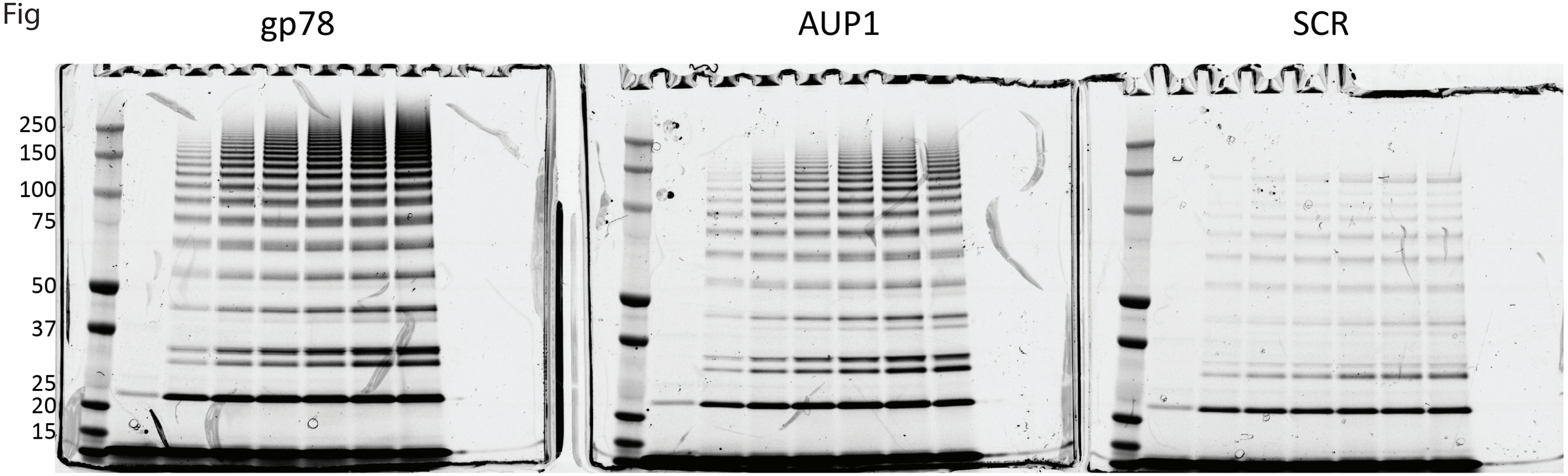

S5B Fig

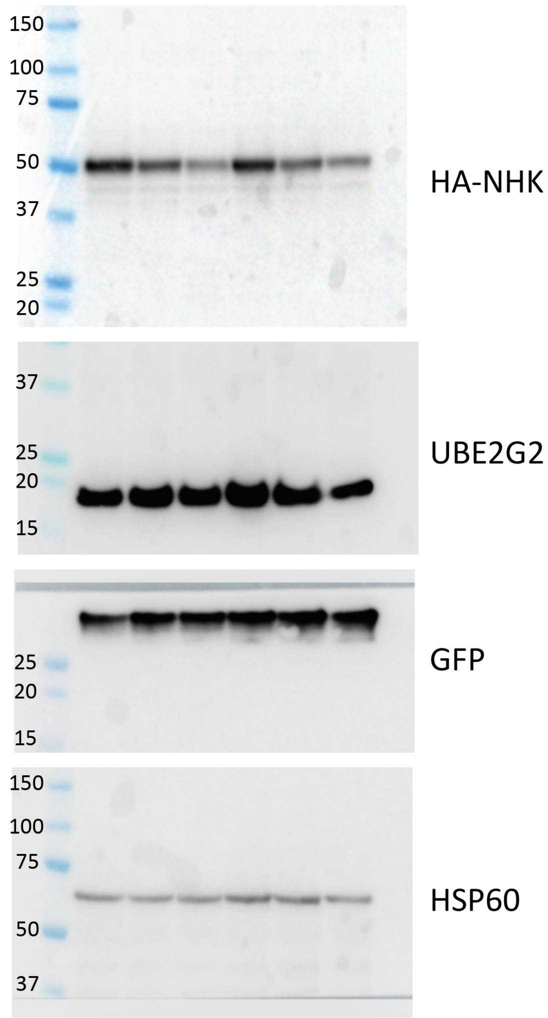

S5C Fig

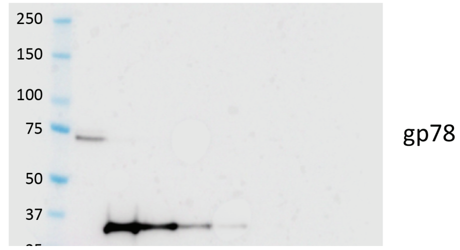

S5D Fig

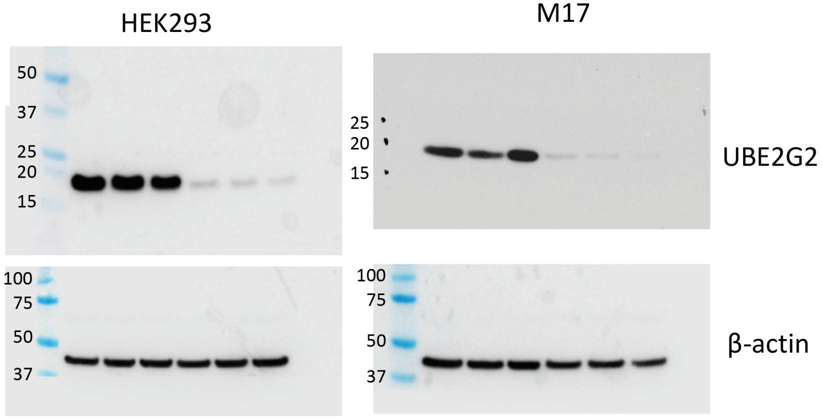

S5E Fig

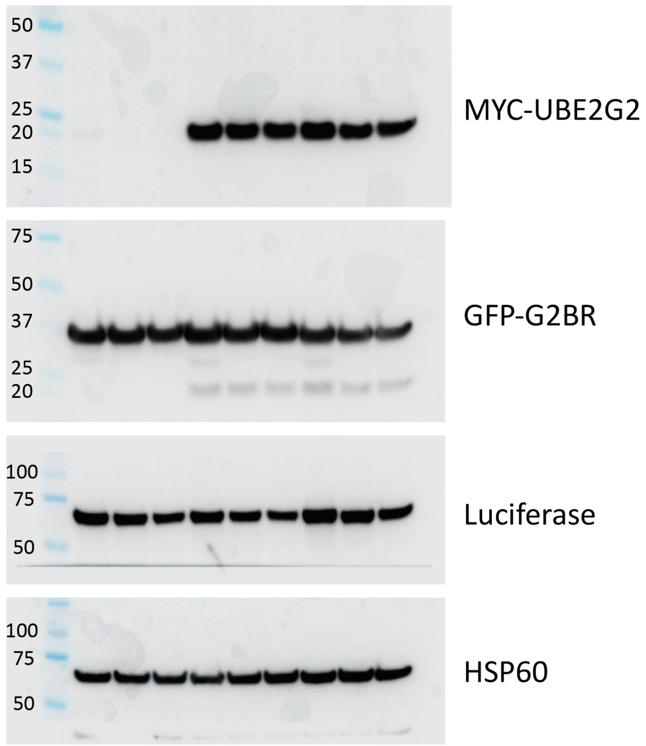

S5F Fig

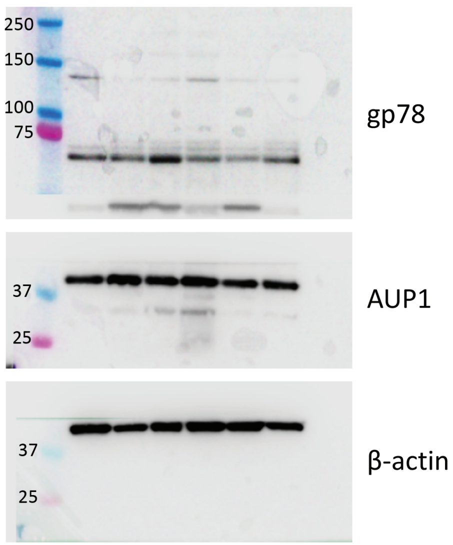

S6A Fig

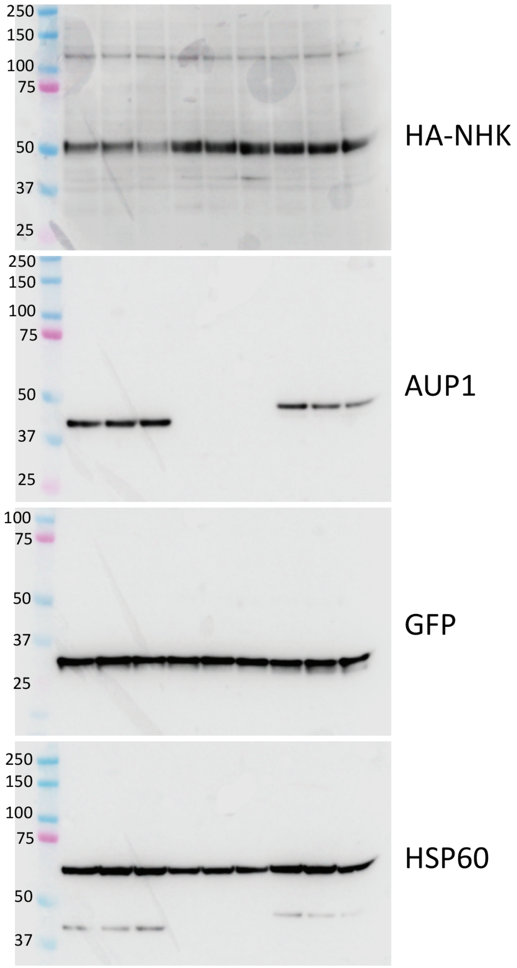

S6B Fig

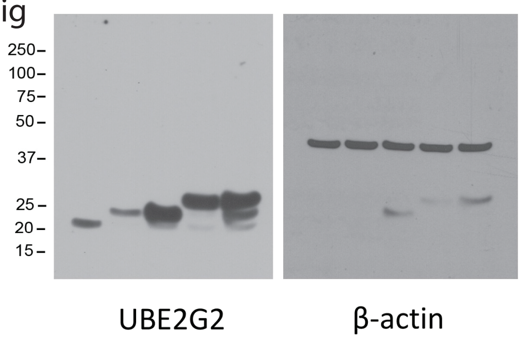

S6D Fig

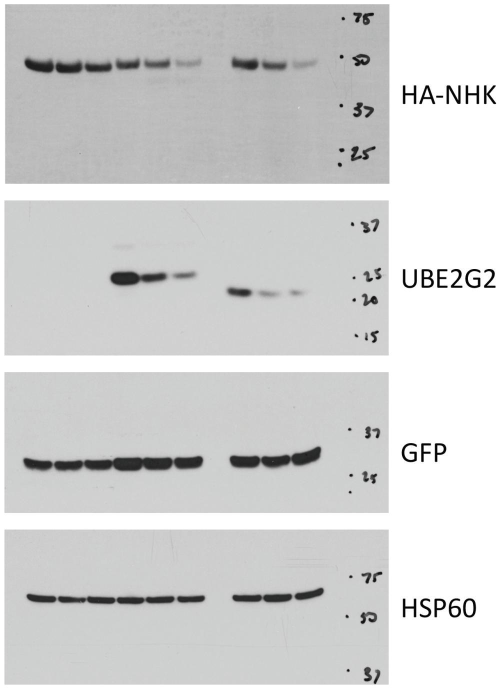

S6C Fig

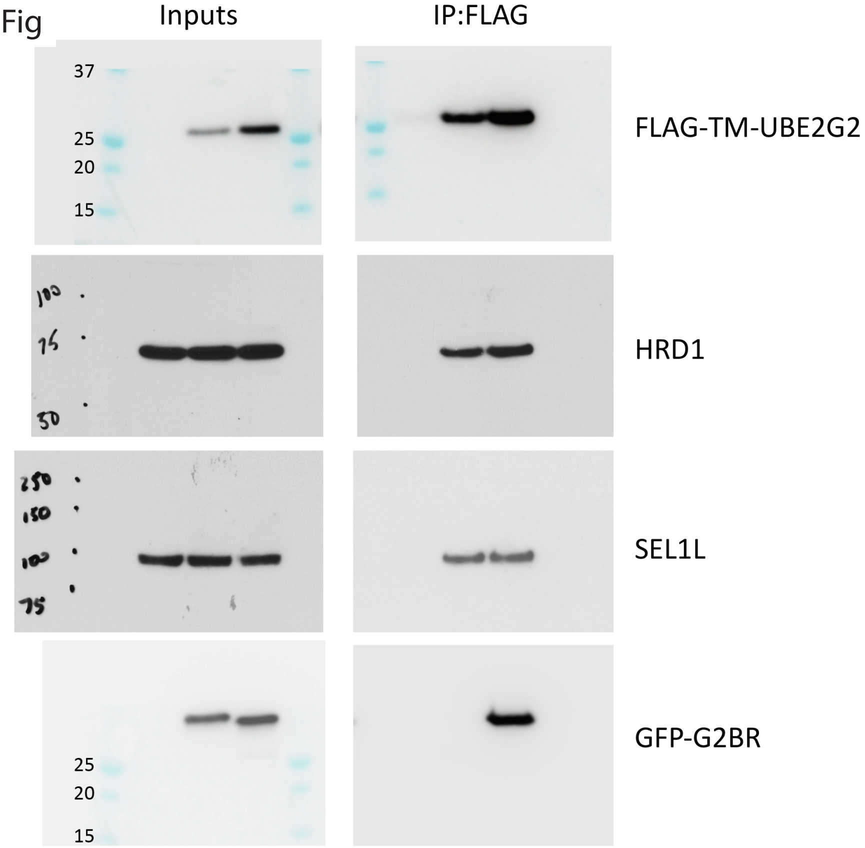

Supplement: S2 Data — (PDF) [file pbio.3001474.s008.pdf]
