# Supplementary material for: A structurally conserved site in AUP1 binds the E2 enzyme UBE2G2 and is essential for ER-associated degradation
Source: PLoS Biol. 2021 Dec 8;19(12):e3001474. doi: 10.1371/journal.pbio.3001474 (PMC8699718; doi:10.1371/journal.pbio.3001474)
Supplement: S1 Table — (PDF) [file pbio.3001474.s015.pdf]

S1_Table

Primary data values for the plots in Figure 5 D,E. The data are reported as the ΔCSP (A,C), which is setting the initial shift with no G2BR at 0.0, and reporting the computed ΔCSP from this reference value. The data are also reported as $\Delta CSP/\Delta CSP_{max}$, which is normalized (B,D) to the maximum ΔCSP observed at a ratio of 1:3.0 for (Ube2G2:G2BR):RING. Table A, B are for titrations of (UBE2G2:G2BR^{AUP1}) [1:1.1] and Table C,D are for titrations of (UBE2G2:G2BR^{gp78}) [1:1.1]. Values reported are from experiments conducted at a field strength of 600 MHz for ^{1}H.

A.

**AUP1-G2BR^{AUP1}**	ΔCSP	(ppm)				
(UBE2G2:G2BR):RING ratio	0	0.33	0.67	1.0	2.0	3.0
E12	0	0.0335	0.0628	0.0755	0.1101	0.1189
Q15	0	0.02943	0.0544	0.0766	0.1024	0.1183
L66	0	0.0633	0.1067	0.128	0.1661	0.1916
V113	0	0.0270	0.0547	0.0735	0.1018	0.1025

B.

**AUP1-G2BR^{AUP1}**	$\Delta CSP/\Delta CSP_{max}$	(ppm)				
(UBE2G2:G2BR):RING ratio	0	0.33	0.67	1.0	2.0	3.0
E12	0	0.2817	0.5282	0.6350	0.9260	1.0000
Q15	0	0.2488	0.4598	0.6475	0.8656	1.0000
L66	0	0.3304	0.5569	0.6681	0.8669	1.0000
V113	0	0.2634	0.5337	0.7171	0.9932	1.0000

C.

**AUP1-G2BR^{gp78}**	ΔCSP	(ppm)				
(UBE2G2:G2BR):RING ratio	0	0.33	0.67	1.0	2.0	3.0
E12	0	0.0378	0.0628	0.0844	0.114	0.1304
Q15	0	0.0695	0.1063	0.1333	0.1779	0.2016
L66	0	0.0338	0.0562	0.0649	0.10348	0.1105
V113	0	0.0414	0.0673	0.793	0.1107	0.1177

D.

**AUP1-G2BR^{gp78}**	$\Delta CSP/\Delta CSP_{max}$	(ppm)				
(UBE2G2:G2BR):RING ratio	0	0.33	0.67	1.0	2.0	3.0
E12	0	0.2899	0.4816	0.6472	0.8742	1.0000
Q15	0	0.3447	0.5273	0.6612	0.8824	1.0000
L66	0	0.3059	0.5086	0.5873	0.9365	1.0000
V113	0	0.3517	0.5718	6.7375	0.9405	1.0000
