# Supplementary material for: A structurally conserved site in AUP1 binds the E2 enzyme UBE2G2 and is essential for ER-associated degradation
Source: PLoS Biol. 2021 Dec 8;19(12):e3001474. doi: 10.1371/journal.pbio.3001474 (PMC8699718; doi:10.1371/journal.pbio.3001474)
Supplement: Attachment — Submitted filename: SmithRevisionResponse_01.pdf [file pbio.3001474.s016.pdf]

A structurally conserved binding site for UBE2G2 in ancient ubiquitous protein 1 (AUP1) is essential for endoplasmic reticulum-associated degradation (ERAD)

Response to Reviewer Comments

We thank all three reviewers for their extremely helpful suggestions, which have made for an improved manuscript. Before getting into specific responses, we provide below a list of additions that we have made to the manuscript.

Overall:

- Data originally corresponding to Fig 1 has been expanded and is now divided between Fig 1 (NHK) and the new Fig 2 (RI_{332} and GFPu), making for eight figures in the main text.
- New Fig S5 is composed entirely of new data largely corresponding to Fig 7 (formerly Fig 6). The former Fig S5 is now Fig S6.

Specific additions in response to the reviews:

- Expansion of our central findings into HEK293 cells, where we have now knocked out AUP1 (Fig 1G, Fig 1H, Fig 2C, Fig 2D, Fig S5D, and Fig S5E). Data from an M17 AUP1 knockout are also provided in Fig S5D. All of the results are consistent with our original findings in HT1080.
- Expansion of AUP1 domain requirements to an ERAD-M substrate (HMG-CoA Reductase) (Fig S1D and Fig S1E).
- An experiment demonstrating a requirement for the G2BR of gp78 in the degradation of INSIG-1, an ERAD-M substrate, is shown in Fig S1A. This was not specifically requested by any of the Reviewers, but we believe it helps to round out the story.
- Immunofluorescence showing the ER localization of TM-UBE2G2 in HT1080 (Fig 8F).
- Establishment of a role for HRD1 in RI_{332} degradation (Fig 2A).
- Demonstration that TM-UBE2G2 works with soluble G2BR to restore ERAD of RI_{332} (Fig 8I).
- Co-IP of TM-UBE2G2 with HRD1 and with SEL-1L (Fig S6C).
- Assessment of the relative levels of AUP1 and gp78 across five additional human cell lines (Fig S5F).
- Level of GFP-G2BR relative to endogenous gp78 (Fig S5C).
- Demonstration that loss of UBE2G2 evokes a ER stress response/UPR that accounts for the increase in HRD1 (Fig S2C and S2D).
- Removal of ubiquitin from HRD1 by DUBs (Fig S2F).
- Time course experiments showing increased processivity of ubiquitination *in vitro* in the presence of G2BRs (Fig S4B).
- Loading of cells with oleic acid showing no discernable effect on either NHK degradation or UBE2G2 stability (Fig S5A and Fig S5B).

The complete comments from each Reviewer and a point-by-point response (in blue) are presented below:

Reviewer #1: The manuscript submitted to PLoS Biology by Weissman and colleagues reports on a long awaited analysis of the mechanism underlying the role of AUP1 in ERAD, and more specifically on its molecular activation of partner E3 ubiquitin ligases via an embedded G2BR.

A related domain, U7BR was previously analyzed by this group as a "back-side" binding motif in yeast Cue1 for a partner ubiquitin conjugating enzyme, Ubc7. In addition, in the mammalian E3, gp78, a similar motif, G2BR, bound and activated an analogous ubiquitin conjugating enzyme, UBE2G2. Interestingly, a Cue1 analog in higher cells, AUP1, has been implicated in not only the ubiquitination of some ERAD substrates (although its substrate specificity is somewhat controversial), but in lipid droplet homeostasis by virtue of an acyltransferase activity. The CUE domain may interact with both ubiquitinated substrates as well as lipids. Similar to what was observed for Cue1 and gp78, AUP1 also interacts with UBEG2 via an embedded G2BR, but the role of this motif and the acyltransferase moiety in ERAD, let alone other AUP-associated functions, has not been investigated.

To this end, Smith et al. first find that AUP1 is required for the degradation of three diverse ERAD substrates, an effect that depended on G2BR but not on the acyltransferase or CUE1 domains in AUP1. As expected, the ubiquitination of one substrate (but not HRD1) also required the domain. The authors next investigated the specificity of G2BR from AUP1 and the homologous G2BR in gp78. The G2BR from AU1P bound in vitro translated UBEG2 but neither UBEG1 (which binds the gp78 G2BR) nor several "promiscuous" E2s. To further investigate the mechanism underlying UBE2G2-G2BR specificity and function, the structure of this complex was solved by X-ray crystallography. As anticipated, the structures of the G2BRs from AUP1 and gp78 were similar and a high affinity (~3 nM) interaction, mediated by select amino acids, with UBE2G2 was measured. NMR spectroscopy indicated that the G2BR from AUP1, like that from gp78, alters the conformation of the E2. Based on the measured affinity of the G2BR and comparisons to RING domain association with the E2, the resulting conformational change brought about by the AUP1 G2BR increased the affinity of the E2 for the gp78 RING domain by ~10-fold, an effect that was reflected in enhanced auto-ubiquitinated of two examined (HRD1 and TRC8) ligases. Next, the authors asked whether—as might be anticipated by backside, allosteric activation of the E2 and ligase activity—the G2BR-dependent interaction of UBEG2 with AUP1 plays a role in ERAD. Mutation of 5 polar/charged amino acids in the interaction site abrogated in vitro binding, and similarly failed to support the ERAD of one of the AUP1-dependent substrates (NHK) studied earlier. Mutation of two key hydrophobic residues to Asp had similar effects. A single mutant provided some hint of an intermediate effect on binding (but see below). Based on results from the analogous factors in yeast, Smith et al. then asked if AUP1 protects its partner E2 from degradation. Stability was mediated by either the AUP1 or—more surprisingly—the gp78 G2BR. Nevertheless, UBE2G2 remained stable in gp78 knockout cells, which might arise from its significantly higher levels, at least in HT1080 cells (also see below). Finally, the link between the requirement for AUP1 in HRD1-dependent degradation was investigated. After ruling a potential artefact, the authors focused on the potential role of AUP1 is not only in the stabilization of UBE2G2 but in

recruiting the E2 to the ER membrane. A striking result was obtained when a synthetic ER-tethered form of UBE2G2 was found to rescue AUP1 via G2BR association. These results indicate that yet another role of AUP1 is to harness the E2 to the membrane.

Overall, this is a well written, thorough, and logically presented story that provides new insights into the specific interactions amongst ERAD-associated factors that are required for substrate ubiquitination. The results of this study more specifically support three independent functions of the G2BR in AUP1. The data are exceptionally clean and controlled and utilized diverse techniques. While some of the results were expected based on prior research on the analogous proteins in yeast, there are several striking differences between AUP1 and Cue1 that warrant attention. Thus, the study will be of interest to readers in several fields who use various models.

Comments:

1. In the author's hands, is mutant rhodopsin also a HRD1 substrate (viz. Fig. 1)? In fact, the first sentence of the Discussion indicates that the requirements for the degradation of RI332 were explored in this study, which is not true. In addition to the HRD1 control, the conclusions of this study would be strengthened if select experiments described later in the text utilized this second substrate.

We thank the Reviewer for this important suggestion. We have now confirmed RI_{332} as a HRD1 substrate (Fig 2A) – we mistakenly assumed this was already established in the literature - it appears to only have been surmised based on a requirement for SEL-1L. We have also established a requirement for UBE2G2 in RI_{332} loss (Fig S2A and S2B), which also appears to have not been previously demonstrated. Most importantly, we now provide data that degradation of RI_{332} is reconstituted with a membrane-tethered UBE2G2 + G2BR (Fig 8I). We additionally examine AUP1 domain requirements for this substrate in newly generated HEK293 AUP1 knockout cells (Fig 2C and 2D). Our findings in HEK293 are completely consistent with our observations in HT1080.

2. The data in Figure S4 suggest not only increased auto-ubiquitination by also increased processivity. This effect could be better resolved by showing a time-course.

We now include data demonstrating increased processivity of ubiquitination with addition of G2BRs (Fig S4B).

3. There is a hint from the data in Figure 5D that the A397D mutant, which significantly reduces but does not block binding, may allow for limited ERAD of NHK. Since the blots are overexposed and only a single experiment is shown, have the authors thought about examining whether binding and the associated support of ERAD is a titratable phenomenon?

We have gone back and looked at our binding experiments and don't see anything that would suggest any significant binding to A397D above background, even at the most sensitive settings. We also realized that we had omitted the fact that GST-AUP1 fusions are present at ~15-fold molar excess relative to the E2 in these binding assays, information on the amount of labeled E2 has been added to Methods. We have also repeated the transfection experiment as suggested,

doubling the amount of A397 (this is the most we can transfect without adverse effects from too much plasmid). We are unable to convince ourselves that there is any significant degradation beyond that seen with control vector transfection.

4. A better test of the model that the levels of gp78 relative to AUP1 dictate which G2BR-containing protein stabilizes UBE2G2 (Figure 6) is to examine the levels of the two proteins in other cell lines. More likely than not, perhaps the ratio of gp78 to AUP1 differs? Indeed, components involved in cellular quality control vary widely between cells.

We have surveyed lysates from five additional cell lines (Fig S5F). While there is some variation, interestingly a marked greater prevalence of AUP1 compared to gp78 is a consistent theme.

Minor comments

5. Since the ubiquitination pattern of HRD1 is clearly delineated as starting at ~75 kD (Fig. 2F), the authors should note the molecular mass of this E3.

The molecular mass of HRD1 is now noted in what is now Fig 3F.

6. In the relevant section of the text, the reader should be referred to the affinities calculated using FP, which are presented in Figure 3D.

We have changed where we refer to Fig 4D (previously Fig 3D) as well as to the corresponding supplemental figures.

7. The x-axis in Figure 4E lacks a label.

The x-axis of Fig 4E (now Fig 5E) is [RING]/UBE2G2:G2BR. We have clarified this in the figure legend.

8. Without explanation, it appears that U2OS cells were used for the experiment in Figure 7D. What was the rationale for this switch? The data linking the results in Figure S5 (in HT1080 cells) to this figure would best to compared with identical cell types.

The switch in cell types was done to employ a cell that lends itself to high resolution visualization of the ER. We now provide confocal images of HT1080 cells (Fig 8F). We have retained the U2OS data (now Fig 8G) as these SIM images provide a higher resolution view.

9. Perhaps Figures S5C-D, which provide key verification of the chimera, could accompany the results within Figure 7.

As suggested, Fig S5C and S5D have been relocated to the main text as Fig 8D and 8E.

Reviewer #2 (John C Christianson): MS #: E17-08-0514

TITLE: A structurally conserved binding site for UBE2G2 in ancient ubiquitous protein 1 (AUP1) is essential for endoplasmic reticulum-associated degradation (ERAD)

AUTHORS: Smith et al.

OVERVIEW

In this manuscript, the authors describe a multifaceted role for the G2BR binding site of AUP1 in ER-associated degradation (ERAD). They build on a variety of data from their lab and other reports to characterise this domain in detail and provide new insights into the role that this interaction might be playing in ERAD. Confirming screening data reported previously, the authors confirm essential roles for both AUP1 and Ube2g2 in turnover of ERAD-L (NHK & RI332) and ERAD-C (GFPu) substrates using CRISPR KOs. The authors demonstrate ERAD of model substrates can be rescued by full length AUP1 and almost all truncations/deletions/mutants that do not disrupt the G2BR or disconnect it from the ER membrane. Ubiquitination of substrates but not the E3 Hrd1 is attenuated in KOs of both AUP1 and Ube2g2, leading the authors to differentiate its role from that of another E2, Ube2j1. The authors use recombinant proteins and in vitro assays to show specificity of Ube2g2 for AUP1 and its G2BR. ΔUbe2g2 but not ΔAUP1 cells exhibit defects in degradation of Insig1, a gp78-dependent membrane protein. The authors also provide NMR data which supports a finding where the G2BR domain in AUP1 behaves comparably to that found in gp78, going on to identify residues supporting key electrostatic and hydrophobic interactions between AUP1 and Ube2g2. AUP1 variants containing mutations to key residues in the G2BR responsible for Ube2g2 binding as determined by NMR, lost AUP1 binding and were not able to rescue NHK degradation. Like the G2BR-gp78, the authors find, perhaps not unexpectedly, that G2BR-AUP1 also activates Ube2g2. Interestingly, they do find that interaction with AUP1-G2BR rather than gp78-G2BR has prominent role in protecting Ube2g2 by maintaining its stability and keeping it associated with the ER membrane. The authors attribute this to a ~40-fold higher abundance of AUP1. The authors provide evidence that Ube2g2 minimally requires the high-affinity G2BR binding and ER recruitment to functionally restore ERAD, with the acyltransferase and upstream ubiquitin-binding CUE domain, entirely dispensable. These data lead the authors to conclude a multifaceted role for AUP1 and its G2BR - to activate Ube2g2, to keep it from self-targeting for degradation, and to recruit it to the ER membrane, collectively serving to ubiquitinate ERAD substrates.

Overall the manuscript is suitable in detail and scope for the journal. While not presenting entirely novel concepts, what the authors have done is to put together a thorough collection of experiments that clarifies a lot of previous data to yield a clearer picture of the role that AUP1, its G2BR and Ube2g2 play in ERAD. There are several intriguing implications from these data, which will help to guide the field to a better understanding of the ERAD mechanism, while also providing general appeal to an audience keen to understand complex, regulated ubiquitination

mechanisms. The manuscript is clearly written and appropriately referenced. In this reviewer's view, there are just a few issues that could require attention.

SELECTED ISSUES

Issue 1. The authors provide data demonstrating the ERAD-L substrates NHK and RI332 require both AUP1 and Ube2g2 and Hrd1, while Insig-1, an ERAD-M substrate only requires Ube2g2 and gp78. The clear omission here is the absence of a Hrd1-dependent ERAD-M substrate to directly confirm the AUP1-Ube2g2 roles in ERAD. The breadth of the conclusions would be greater if this could be shown, perhaps simply by probing for endogenous CD147 and monitoring its ER-resident form (see Tyler et al. JBC. 2011 or Schulz et al. JCS 2017) or MHC Class I with B2m silencing (see Burr et al. PNAS 2011).

We thank the Reviewer for this suggestion. In Burr et al., the degradation of the GFP-Class I fusion is dependent on luminal determinants as the requirements for degradation of transmembrane and non-transmembrane forms is identical. Thus, even though Class I has a TMD, it should, if anything, be defined as an ERAD-L substrate. We have looked at CD147 and found that effects of AUP1 loss on the maturation/degradation of this protein to be relatively minor and not consistent across cell lines, but of course the dynamics of assembly/maturation vs. degradation are not easily sorted out. More importantly, however, we have found that re-expression of AUP1 in AUP1 knockout cells, or overexpression of AUP1 in WT HT1080 can lead to a paradoxical increase in immature CD147 (see example below showing WT HT1080). This suggests that AUP1 is having effects on other proteins involved in the complex maturation of this protein. This is interesting, but clearly beyond the scope of this study.

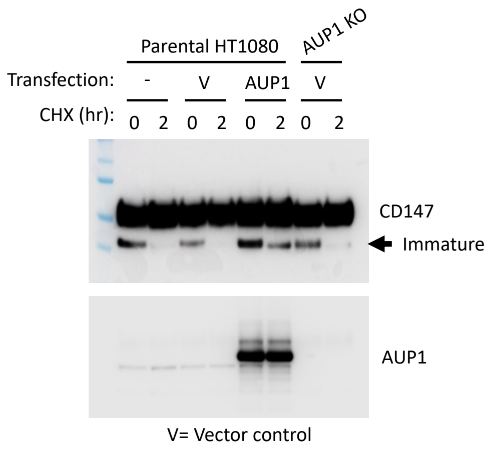

In yeast, the most well-studied Hrd1p ERAD-M substrate is Hmg2p, and there are data suggesting involvement of HRD1 in the slow basal degradation of the mammalian ortholog, HMG-CoA reductase (Kikkert et al. 2004). Therefore, in an attempt to address the Reviewer's question, we have examined the role of AUP1 in the basal degradation of endogenous HMG-CoA reductase. The data we obtained now extends the specific requirement for the G2BR^{AUP1} to the basal degradation of HMG-CoA reductase. These data are now provided in Fig S1D and S1E. We did not revisit ligase requirements for this degradation (and do not claim this degradation is mediated only by HRD1), as we are quite aware that the literature on the lipid-stimulated

degradation of this protein is quite 'complex' (at least four E3s have been implicated). It is likely that other E3s could be implicated in the basal degradation as well. Nonetheless, this establishes a specific requirement for $G2BR^{AUP1}$ in the degradation of an ERAD-M substrate.

To further expand on the requirements for G2BRs in ERAD-M, we also now include data demonstrating a requirement for the $G2BR^{gp78}$ in degradation of INSIG-1. We had previously shown that self-degradation of gp78 requires its G2BR but had not explicitly addressed a requirement for this domain for a heterologous substrate. These data are now included in Fig S1A.

Issue 2. In line 335, the author ascertain that there is nearly 40-fold more AUP1 relative to gp78 in HT1080 cells. This claim prompts several questions. First, is the amount of GFP-G2BR expressed to protect Ube2g2 in ΔAUP1 cells (Figure 6D) near to the ~40x amount of endogenous gp78?

We now include data assessing the level of GFP-G2BR that is typically expressed in our experiments relative to endogenous gp78 (Fig S5C). The overall level of overexpression is in the range of ~40-fold that of endogenous gp78.

Secondly, Figure S5C shows increased cytosolic partitioning of Ube2g2 in ΔAUP1 cells compared to Δgp78 or WT, but this amount does not appear to be 40x different as might be expected, and more cytosolic. Does this imply that Ube2g2 levels might be rate limiting in normal cells, with competition between gp78 and AUP1 for binding? Perhaps the authors could comment on this.

If we understand correctly, the Reviewer is asking why (referring to what is now Fig. 8D), in AUP1 KO cells, there is not 40-fold more cytoplasmic UBE2G2 than membrane-bound UBE2G2 due to the loss of AUP1 and the retention of only gp78 as a source of G2BRs. As UBE2G2 is unstable in the absence of AUP1, its levels in AUP1 KO cells are therefore markedly lower. Another consideration in assessing this data is that we have no idea whether there are other UBE2G2 binding domains besides the RING domains of ERAD E3s. And for RING domains, we don't know whether the low affinity in vitro binding would be altered in the cellular context with UBE2G2~Ub. With regard to the question of whether UBE2G2 could be rate-limiting for ERAD in normal cells, there is nothing in the experiments we have carried out, or could carry out within the scope of this study, which would provide an answer to this question. However, it is certainly possible, if not likely, that AUP1 can have a rheostat-like function. Depending on the cellular context it might either limit or enhance the amount of UBE2G2 that is available for ERAD. We have now made explicit mention of this idea in the Discussion and thank the Reviewer for encouraging us to expound on the implications of AUP1's regulation of UBE2G2 levels.

Issue 3. In Figure 7, the authors provide data supporting the idea that mere tethering of the Ube2g2 to the ER membrane (TM-UBE2G2) via an unrelated TMD along with a soluble AUP1-G2BR is sufficient to restore Hrd1-dependent degradation of NHK. Are the authors implying that

interaction/s of AUP1 with the Hrd1 complex are dispensable for this process? This would be slightly surprising to this review so perhaps the authors might expand further on the implications here.

To address the Reviewer's question, we generated a C-terminally FLAG-tagged version of the chimera that could be efficiently IP'ed and found that, like AUP1, it associated with SEL-1L and HRD1 under conditions where the AUP1 association had been observed. We also found that this occurred independent of the co-expression of the G2BR (Fig S6C).

SPECIFIC COMMENTS/QUESTIONS

Comment 1. For the ubiquitination assays, the authors use concentrations of MG132 that are unexpectedly high (e.g. 40µM) and well into the range of non-selective effects. Is there a reason why better ERAD inhibitors, such as CB-5083 (@ 10nM) or bortezomib (@25nM), were not used?

We very much appreciate the Reviewer's question. When our goal is to assess mechanism of degradation we use "specific" inhibitors, and never rely solely on MG132, even at a low dose. However, the goal of the ubiquitination assays was not to probe the mechanism of degradation, but rather to examine protein ubiquitination and to stabilize proteins that have been ubiquitinated. Regarding the concentration of MG132, the amount to which cells are subject in the ubiquitination assays is 25 µM, which is within the range that is generally used for short term treatment of cells. 40 µM was included in cell lysis buffer in some experiments to mitigate possible post-lysis protein degradation.

Comment 2. In Figure 2E, ER stress induction is proposed as the reason for increased Hrd1 levels in Ube2g2 KO cells, but this is not directly demonstrated. Would it be possible to perform a simple XBP-1 splicing assay to confirm this or to also probe for another ER stress regulated gene such as Herp or Bip? This would help to solidify this point.

We now demonstrate that loss of UBE2G2 results in induction of cellular stress as assessed by multiple protein markers and by qPCR (Fig S2C-D).

Comment 3. Previous studies have reported AUP1 to be mono- and di-ubiquitinated (Klemm et al. JBC 2011), yet this form does not appear to be present in any of the western blots presented. Could the authors address why this discrepancy might exist?

We thank the Reviewer for this question.

i) We neither assert that AUP1 is not ubiquitinated nor proffer whether specific AUP1 domains may play a role in such a modification.
ii) Our experiments, including cell lysis conditions used for visualizing AUP1, were not designed with the goal of visualizing ubiquitinated AUP1.
iii) Klemm et al. does not experimentally address the significance of the ubiquitination that they observe. In particular, the NH2-GFP-AUP1-COOH protein product, for which ubiquitination was most notable, is not functionally characterized in their study.

Thus, from a functional perspective, we do not believe there is a specific discrepancy to address.

Comment 4. The authors present Hrd1 ubiquitination data for both transfected and endogenous forms, which is notable in that smears do not appear to change under any conditions. This reviewer would feel more reassured with these data if smears could be at least shown to be sensitive to recombinant DUB treatment or lost from cells where Ube2j1/Ubc6e is knocked down or out, as has been suggested by other data in the manuscript.

A demonstration that the higher molecular weight species can be captured with ubiquitin-binding domains and subsequently removed with DUB treatment is now provided (Fig S2F). We did initiate attempts to develop UBE2J1 knockout cells, but to date we have been unsuccessful.

Reviewer #3: The article by Smith, C. E. et al., describes an important role for ancient ubiquitous protein 1 (AUP1) and its interaction with the E2 conjugating enzyme, through its C-terminal G2BR domain, in ER-associated degradation (ERAD). Using a fibrosarcoma cell line (HT-10180), the authors initially describe the requirement for AUP1 in HRD1-mediated degradation of the Null Hong Kong (NHK) variant of alpha1-antitrypsin. They also provide evidence, through deletion mutations, that AUP1's most significant contribution to the degradation of ERAD substrates is from its UBE2G2 binding region (G2BR) and that the acyltransferase and Cue1 domains are dispensable. Membrane and luminal ERAD of INSIG1 and NHK (respectively) is shown to require functional UBE2G2 for ubiquitination (NHK and HRD1) and turnover (NHK and INSIG1).
The authors elegantly define the interaction between UBE2G2 and AUP1 using crystallography, mutation of residues and biochemistry. While comparing the structure and interaction of UBE2G2 and AUP1's G2BR domain they also highlight how similar the interaction and kinetics are to that of gp78's, G2BR interaction with UBE2G2. The authors report that many of the amino acid residues required for the interaction of AUP1 and UBE2G2 are hydrophobic and show that three specific residues L386, Y394 and A397, when mutated to aspartic acid, prevent the interaction between UBE2G2 and AUP1.
The interaction of AUP1 with UBE2G2 appears to stabilise levels of UBE2G2 in the HT-1080 cell line and only deletion of AUP1 and not gp78 de-stabilises UBE2G2. This result is not due to significant changes in the mRNA levels but does require UBE2G2 activity - catalytically dead C89S UBE2G2 does not exhibit the same phenotype. Confusingly, addition of GFP-tagged gp78 or AUP1 G2BRs resulted in the stabilisation of UBE2G2. The authors reconcile this data by showing that AUP1's role in UBE2G2 stability is a result of higher levels of expression compared to gp78. Finally, they show that AUP1 is dispensable if a transmembrane localised UBE2G2 is expressed alongside GFP-tagged AUP1 G2BR. Taken together this suggests the minimum requirements for NHK turnover are an ER membrane bound protein that can interact with or is itself UBE2G2 and a G2BR. If either is missing UBE2G2 cannot be utilised in ERAD.
This is a well-planned, executed and expansive paper on the role of G2BRs in gp78 and AUP1 and sheds light on a role of AUP1 in ERAD that has not been previously demonstrated. There are a few issues that arise that could be address to make this paper much stronger and make it more accessible to a larger readership.

Major points:

The phenotypes they show are strong but it would be nice to see the initial observations repeated in other cell lines.

We thank the Reviewer for suggesting we extend our results to other cell lines. A lot of work, but we believe this very significantly enhances the impact of this study. We have now generated HEK293 AUP1 KO cell lines and have recapitulated our initial results on domain requirements for both NHK and RI_{332} (Fig 1G-H, Fig 2C-D). We also provide data that E2 loss similarly occurs in these HEK293 AUP1 KO cells and that E2 stability can be restored by GFP-G2BR (Fig S5D and S5E). To extend our observations beyond HT1080 and HEK293, we also generated and examined M17 AUP1 KO cells, and observed a similar destabilization of UBE2G2 with loss of AUP1. We did not pursue additional studies in M17 cells as they are significantly more difficult to manipulate.

Although, it is possible that the stability of UBE2G2 is due to the level of AUP1 in these cells, it is not clear whether the G2BR of AUP1 is always available for UBE2G2 binding and whether AUP1 acts as a sink for UBE2G2 when associated with lipid droplets. As the authors state, AUP1 has multiple roles in cells, and therefore a clear experiment that is needed here is immunoprecipitation of endogenous UBE2G2 followed by a blot for AUP1 and gp78 to show that they bind different levels of UBE2G2 under the conditions used by the authors in these experiments. They can then compare the 'pulldown levels' of AUP1 and gp78 to the amounts of protein in the reference binding domains in (Figure 6).

It would also help their assertion that 'more AUP1 equals more UBE2G2 due to G2BR binding', if they could show that AUP1 binding can be titrated away from UBE2G2 when cells are fed increasing amounts of Oleic acid, or whether this interaction is unaffected as AUP1 is re-localised to lipid droplets. Alternatively, NHK degradation may be reduced under increasing Oleic acid levels but UBE2G2 binding is not affected. This would provide further evidence that AUP1 is indeed a protective sink for UBE2G2 under multiple conditions and that protein levels are a significant reason for why AUP1 is important for preventing UBE2G2 turnover.

We have now loaded cells with oleic acid and assessed E2 levels and NHK degradation (Fig S5A and Fig S5B). Neither of these were significantly altered. Thus, alterations in lipid droplets does not appear to alter the availability of AUP1 for ERAD, at least by these parameters. Our negative findings do not, of course, mean that there are not situations where lipid droplets affect ERAD. Therefore, in describing our findings we specifically state that there is no effect "in our system".

Assessment of the relative co-IP of endogenous AUP1 and gp78 with endogenous UBE2G2 is not practical as the antibodies against this E2 will not allow for IP under non-denaturing conditions. We have examined co-IP of the two proteins with transfected MYC-UBE2G2 +/- oleic acid. Based on three experiments using WT HT1080, gp78 appears to be co-IP'ed about twice as efficiently as AUP1. However, as gp78 binds through both the RING and the G2BR, the overall avidity of gp78 for UBE2G2 would be predicted to be substantially greater than AUP1, at least when AUP1 is extracted from the ER membrane and associated proteins. Thus, it is not

evident that there is a straightforward interpretation of these results. In these experiments, oleic acid loading reduced the efficiency of co-IP of both gp78 and AUP1, with a decrease of gp78 co-association of ~20% and AUP1 co-association of ~27%. Again, it is not evident that we can ascribe any significance to this, particularly as there is no evidence that oleic acid affects levels of endogenous UBE2G2 or the degradation of NHK (Fig S5B). Nonetheless, we are appreciative to the Reviewer for suggesting we examine whether AUP1 partitioning to lipid droplets could have functional consequences for ERAD.

Although U2OS cells provide nice results when looking at ER and proteins that localise to the ER, the authors have not shown that their mechanism holds in this cell line and do not use them throughout the paper other than to show localisation of their Myc-tagged TM-UBE2G2. Therefore, it is important to either confirm a similar phenotype for these cells or show the distribution of this protein (alongside an ER resident) in the HT-1080 cells.

We have now included IF data for HT1080 cells but have also kept the higher resolution U2OS data (Fig 8F and 8G).

Minor points:
It is unclear why the GFP/Luciferase blots are included as transfection controls. Unless the GFP is upstream or downstream of the protein of interest or connected via a viral P2A sequence it will not directly report on how well a transfection has occurred for the protein of interest. I cannot see a reason for including this control in most blots and in several it probably suggests there is clear disparity between 2 cell types even though the protein(s) of interest are expressed at quite similar levels.

It is our experience that when comparing levels of a transfected protein within a cell line that, absent a viral P2A or the equivalent, it is standard practice to include plasmids encoding stable proteins as transfection controls, especially when assessing stability or accumulation of a protein substrate. It is also our experience that when these controls are not included, they are often requested by reviewers. Thus, we include these whenever possible.

We hope that the reviewers will now find this study suitable for publication.

Sincerely,
Allan Weissman (on behalf of the authors)
